# Anatomical, taxonomic, and phylogenetic reappraisal of a poorly known ghost knifefish, *Tembeassu marauna* (Ostariophysi: Gymnotiformes), using X-ray microcomputed tomography

**DOI:** 10.1371/journal.pone.0225342

**Published:** 2019-11-27

**Authors:** Luiz A. W. Peixoto, Aléssio Datovo, Ricardo Campos-da-Paz, Carlos D. de Santana, Naércio A. Menezes

**Affiliations:** 1 Museu de Zoologia da Universidade de São Paulo, Seção de peixes, São Paulo, SP, Brazil; 2 Universidade Federal do Estado do Rio de Janeiro, Centro de Ciências Biológicas e da Saúde, Instituto de Biociências, Rio de Janeiro, RJ, Brazil; 3 National Museum of Natural History, Smithsonian Institution, Division of Fishes, Department of Vertebrate Zoology, Washington, DC, United States of America; Pontificia Universidade Catolica do Rio Grande do Sul, BRAZIL

## Abstract

A detailed osteological study of the poorly known and critical endangered ghost knifefish, *Tembeassu marauna*, from the rio Paraná, Brazil, was conducted using X-ray microcomputed tomography (μCT scan). A redescription of the external anatomy was performed, including the unusual presence of a rostral patch of extra teeth on the region of the upper lip anterior to the premaxilla and the prominent anterior fleshy expansions in both upper and lower lips. The newly surveyed characters were included and analyzed in light of a recent morphological data matrix for Gymnotiformes. In spite of some uncertainties that remains as to phylogenetic allocation of the genus, the most probable hypothesis is that *Tembeassu* is the sister group of a clade that includes *Megadontognathus* and *Apteronotus sensu stricto*. The phylogenetic analysis also supports that *Tembeassu* is considered a valid genus of Apteronotidae. An amended diagnosis for the genus is also provided.

## Introduction

Ghost knifefishes are members of the Neotropical electric fishes family Apteronotidae, a species-rich clade with more than 100 described species included in 15 genera [1]. Apteronotids can be promptly recognized among gymnotiforms by the presence of a caudal fin and a dorsosagittal electroreceptive fleshy filament [2–5].

In Central and South America, apteronotids inhabit rivers channels, streams, and rapids [6–9]. They are particularly abundant in both number of species and biomass in deep-water channels (> 5 meter depth, e.g., [6,7,10,11]). In those habitats, apteronotids are an important component of the ecosystems where channel-restricted catfishes frequently feed heavily on them. For instance, species of *Brachyplatystoma* are known to prey on *Adontosternarchus*, *Apteronotus*, and *Sternarchorhamphus* [12].

As in all gymnotiforms, species of Apteronotidae generate electric organ discharges (EODs) for navigation and electrocommunication [13,14]. EODs of ghost knifefishes typically have frequencies higher than those generated by other gymnotiforms, ranging from 421 Hz in *Orthosternarchus* to 2,179 Hz in *Sternarchella* [15].

Morphological diversity observed in Apteronotidae can be in many instances associated with trophic specializations or secondary sexual dimorphism. For example, *Sternarchella duccis* (Lundberg, Cox-Fernandes & Albert) have upturned mouth with conical teeth to feed on caudal filament of other gymnotiforms [16]. In the same way, members of *Sternarchorhynchus* use their minute mouths with conical teeth and tubular snouts for grasping and sucking invertebrate larvae and aquatic insects hidden in logs and other substrates [17,18]. Adults of *Adontosternarchus*, in turn, have no oral teeth (e.g., [19]) and prey on aquatic insects in the bottom of rivers [17]. Apteronotids are characterized by the presence of extreme cases of secondary sexual dimorphism in males manifested in at least three different ways [20]: 1, hypertrophy of the preorbital region in males of species in *Apteronotus*, *Compsaraia*, and *Parapteronotus* [21–24]; 2, hypertrophy of the premaxilla and dentary teeth in *Sternarchogiton* [21]; and 3, hypertrophy of dentary teeth in *Sternarchorhynchus* [8].

The poorly-known monotypic *Tembeassu* Triques was established on the basis of only three specimens collected from a coffer-dam during the building process of the Ilha Solteira reservoir at rio Paraná (Ilha Solteira, Mato Grosso do Sul State, Brazil) in 1965. Subsequent collection efforts failed in collecting additional specimens unequivocally assigned to its type species, *T*. *marauna*. The taxon was originally diagnosed by the presence of a single external morphological feature, namely, “an enlarged fleshy lateral lobe on the chin” that would constitute an “autapomorphy” [25]. No characters related to internal anatomic systems (*e*.*g*., osteology) were investigated by Triques [25].

Despite the scarcity of information on *T*. *marauna* and the non-inclusion of this taxon into his data matrix, Albert [4] allocated the species in the “*Apteronotus sensu stricto*” (his “Clade Z”), a group defined mainly on the basis of osteological characters. The same author later unjustifiably listed *T*. *marauna* under *Apteronotus* Lacépède ([26]: 499). Triques [27] was the first to consider *T*. *marauna* in an objective cladistic analysis, *i*.*e*., including it into a data matrix of an explicit phylogenetic analysis. The primary result of that study was an extensively polytomic strict consensus tree representing the Apteronotidae ([27]: 139, Fig 22), where *T*. *marauna* appears as only distantly related to *Apteronotus* (*i*.*e*., “*A*. *albifrons* + *A*. *jurubidae*”, as restricted by that author in his work) and, as such, the genus was therein considered to be valid. Subsequently, Campos-da-Paz [28] examined radiographs from the type material of *T*. *marauna*, and identified an anteriorly-placed patch of slender conical extra teeth on the upper lip of all specimens (*i*.*e*., loosely attached to soft tissue anterior to the premaxillary bones), a condition apparently unique among gymnotiforms. Also, in that study, Campos-da-Paz discussed some additional characters relevant for both phylogenetic and taxonomic placements of *Tembeassu*. That author further suggested that *T*. *marauna* should be included in the list of endangered species from the upper rio Paraná. The species is currently classified as “critically endangered” in the Brazilian list of threatened and endangered fish and aquatic invertebrate fauna (ICMBio/MMA [29]). Ferraris *et al*. [30] in their checklist of Gymnotiformes listed both the genus and species as valid taxa in the Apteronotidae.

Due the absence of detailed information on the anatomy of *Tembeassu*, that genus has been explicitly excluded from investigations of historical biogeographic or ecological aspects of gymnotiforms, as well as from taxonomic revisions and phylogenetic analyses within the Apteronotidae [23, 31, 32]. The recent improvement and accessibility to high-resolution X-ray micro computed tomography (μCT scan) provided the opportunity to survey, in great detail and in a non-invasive way, the osteology of the type of *T*. *marauna*. Based on newly discovered data, we redescribe this enigmatic taxon, discuss its phylogenetic affinities and taxonomic status within Apteronotidae.

## Material and methods

The present analysis was based on the examination of the three types of the *Tembeassu marauna* (MZUSP 48510, holotype, and MZUSP 23090, two paratypes), which remains as the only known individuals unequivocally belonging to the species. All types were radiographed with conventional X-ray and the holotype was submitted to X-ray microcomputed tomography (μCT scan). This study was carried out under approval of the Animal Care and Use Committee (ACUC) of the Instituto de Biociências, Universidade de São Paulo to A. Datovo (Project #226/2015; CIAEP #01.0165.2014). The research employed only ethanol-preserved specimens deposited in museums and did not involve animal experimentation or fossil examination.

### Meristics and morphometrics

Measurements were taken point-to-point to the nearest 0.1 mm with digital calipers (under a stereomicroscope when necessary), on the left side of all specimens. Measurements and counts follow de Santana & Vari [9] and Peixoto *et al*. [33]. In addition, the following measurements of the mandibular fleshy lateral lobe (= “chin” lobe) were taken: I) “lower-jaw lobe length” (distance between the anteriormost and posteriormost tip of the lobe); II) “lower-jaw lobe width” (largest distance between the lateral and medial margins of the lobe at its widest point); and III) “lower-jaw lobe depth” (distance between the dorsal and ventral margins of the lobe at its deepest point). All measurements are presented as proportions of length to end of the anal fin (*L*_EA_), except for subunits of the head, which are given as proportions of head length (*H*_L_). In fin-ray counts, lower case Roman numerals refer to unbranched rays and Arabic numerals branched rays. Frequencies are given in parentheses after each count and an asterisk indicates counts for the holotype.

### Anatomical terminology and descriptions

Osteological nomenclature follows Fink & Fink [34, 35] and Hilton *et al*. [36], except for the designation of the “displaced hemal spines”, that are based on Albert [4]. The terms “transitional vertebrae” and “anterior vertebrae” follow Hopkins [37] and Campos-da-Paz [38], respectively. The “recurrent ramus of the anteroventral part of the anterior lateral line nerve” follows Carr *et al*. [39] and Vischer *et al*. [40]. Myological terminology follows Winterbottom [41] and Datovo & Vari [42]. In descriptions, the telegraphic style is used only in the topics “External anatomy” and “Color in alcohol” due their descriptive simplicity.

### Radiographs and microcomputed tomography

The holotype of *Tembeassu marauna* was scanned on a 300 kV μ-focus X-ray source microcomputed tomography Phoenix v|tome|x m microfocus (General Electric Company) at the Laboratório Multiusuário de Processamento de Imagens de Microtomografia Computadorizada de Alta Resolução do Museu de Zoologia da Universidade de São Paulo, Brazil. The scan parameters were set to obtain the maximized spatial resolution and better image contrast. To improve image resolution a multiscan of the whole specimens was produced based on three individual scans. X-ray projection images were recorded at 1000 ms of time exposure per image, with 70 kV and 200 mA, 1440 images, and voxel resolution of 27 μm. Reconstruction of raw data was performed using the system-supplied software phoenix datos|x reconstruction v. 2.3.0 (General Electric Measurement and Control Solutions, Wunstorf, Germany). Three-dimensional visualization as well as the analysis of the reconstructed data was performed using VGStudio MAX 2.2.3 64 bit (Volume Graphics GmbH, Heidelberg, Germany). All images were prepared using Adobe Photoshop CS and Adobe Illustrator CS 5.1 (Adobe Systems, San Jose, USA). In the microcomputed tomography plates, different colors were utilized to distinct osteological elements only when the limits between structures were not clearly evident. Figures scaled in 4 mm.

Additional 2D radiographs of all types were obtained with aid of a Kevex, PXS10-16W 130kVp 6 Micron Spot MicroFocus X-Ray Source with end window, Varian PaxScan 4030R Std. GadOx DRZ-Plus Screen, and VIVA k.03 Software.

### Phylogenetic analysis

Parsimony analysis was performed including *T*. *marauna* in a modified version of the morphological dataset available in Tagliacollo *et al*. [31], which includes 221 characters and 167 terminal taxa (S1 File). Explanation of changes performed in the original matrix are presented in Discussion. The character acquisition for the phylogenetic analysis was based in a comparative analysis of the external morphology and osteology.

All multistate characters were treated as unordered. The matrix was then submitted to a maximum parsimony analysis under TNT v.1.5 [43] in a driven search aiming to hit the best score 50 times with 20 iterations of tree-fusing, tree-drifting, ratchet, and sectorial searches [44]. Random seed was set to zero and all remaining search parameters followed program defaults. The most parsimonious trees (MPT’s) obtained in that analysis were then submitted to additional TBR branch swapping in order to maximize the sampling of fundamental trees, up to the memory limit of 99,999 MPT’s. Strict (command “ne*”) and majority (command “ma = 50”) consensuses from all MPT’s retained in the memory were then calculated. As the strict consensus is longer than its fundamental trees, characters with alternative optimizations in different MPT’s should not be directly optimized in that consensus [44]. To prevent this problem, only changes common to all MPT’s were optimized (command “apo[;”) and used to diagnose the clades of the strict consensuses. Inspection of alternative resolutions within Apteronotidae among the retained MPT’s were performed via “Trees > Comparisons > Show resolutions” menu in TNT. We then defined constraints of monophyly for pertinent nodes unique to each alternative allocation of *Tembeassu marauna* (“Data > Define constraints”) and subsequently filtered the tree buffer to retain only the trees fulfilling the defined constraint (“Trees > Tree buffer > Filter”).

### Anatomical abbreviations

Ac, anterior ceratohyal; Ad, anterior displaced hemal spine; Af, anterior cranial fontanelle; Afr, anal-fin rays; An, angulo-articular; Anf, angulo-articular foramen; Bb, basibranchial, Bh, basihyal+basibranchial 1; Bo basioccipital; Br, branchiostegal rays; Cb, ceratobranchial; Ce, vertebral centrum, Cl, cleithrum; Cb, coronomeckelian bone; Cv, caudal vertebra; De, dentary; Dec, dentary canals; Eb, epibranchial; Ec, epicentral; En, endopterygoid; Ep, epioccipital; Es, extrascapular; Ex, exoccipital; Fr, frontal; Hb, hypobranchial; Hd, dorsal hypohyal; Hv, ventral hypohyal; Hy, hyomandibula; Io, infraorbital; In, interhyal; Iop, interopercle; Ip, infrapharyngobranchial; Le, lateral ethmoid; Ma, maxilla; Mc, Meckel’s cartilage; Md, dorsal myorhabdoi; Me, mesethmoid; Mt, metapterygoid; Na, nasal; Na3, third neural arch; Ns, neural spine; Op, opercle; Or, orbitosphenoid; Os, os suspensorium; Pa, parietal; Pc, posterior ceratohyal; Pd, posterior displaced hemal spine; pf, posterior cranial fontanelle; Pfr, pectoral-fin rays; Pm, premaxilla; Po, preopercle; Pp, parasphenoid; Pr, prootic; Pra, proximal radial of anal fin; Prp, parapophysis; Ps, pterosphenoid; Pt, pterotic; Ptp, posttemporal; Qd, quadrate; Ra, radials; Re, retroarticular; Ri, rib; Sc, supracleithrum; Scp, scapula; Sn3, third supraneural; So, supraoccipital; Soc, supraorbital canal; Sop, subopercle; Sp, sphenotic; Sy, symplectic; Tr, tripus; Tsl, superior-lip teeth; Tv, transitional vertebra; Ul, upper lip; Upt, upper pharyngeal toothplate; Ur, urohyal; Ve, vertebra; Vo, vomer.

### Institutional abbreviations

Abbreviations for institutions and collections: FMNH, Field Museum of Natural History, Chicago, USA; MNHN, Muséum National D’Histore Naturalle, Paris, France; MPEG, Museu Paraense Emílio Goeldi, Belém, Brazil; MZUSP, Museu de Zoologia da Universidade de São Paulo, São Paulo, Brazil; USNM, National Museum of Natural History, Smithsonian Institution, Washington D.C., USA. Access to material of these collections and permission for dissections were duly authorized by the respective curators.

### Comparative material examined

Only the HL is exposed in some specimens due the difficulty to access the last of the anal-fin ray; “cs” indicates clear and stained specimens. Apteronotidae: *Adontosternarchus balaenops*: MZUSP 83219 (two of 45, 165.2–175.3 mm *L*_EA_), rio Amazonas, Pará, Brazil. *Apteronotus acidops*: MZUSP 22944 (one, paratype,157 mm *L*_EA_), rio Mogi-Guaçu, São Paulo, Brazil. “*Apteronotus*” *bonapartii*: MPEG 3038 (two of seven, 204.6–217.5 mm *L*_EA_), rio Amazonas, Pará, Brazil. “*Apteronotus*” *brasiliensis*: MPEG 20011 (one, cs, 130 mm *L*_EA_), rio Paracatu, Minas Gerais, Brazil. *Apteronotus camposdapazi*: MZUSP 114134 (one of 13, 120. 7 mm *T*_L_ [regenerated]), rio Tocantins, Tocantins, Brazil. *Apteronotus rostratus*: USNM 317229 (one of three, 142.3 mm *L*_EA_, rio Salado, near Teresita, Colombia. *Compsaraia compsa*: MZUSP 56206 (one of eight, 123.4 mm *L*_EA_), rio Negro, Amazonas, Brazil; MZUSP 56543 (one of eight, 24.7 mm *H*_L_), rio Negro, Amazonas, Brazil. *Orthosternarchus tamandua*: MZUSP 55955 (one of two, 286.3 mm *L*_EA_), rio Amazonas, Amazonas, Brazil. *Sternarchorhamphus mulleri*: USNM 373030 (one of five, 222.2 mm *L*_EA_), rio Negro, Amazonas, Brazil. *Parapteronotus hasemani*: MZUSP 6557 (three, 160–230 mm *L*_EA_), rio Solimões, Amazonas, Brazil. *Platyurosternarchus macrostomus*: MZUSP 105584, (one, 194.2 mm *L*_EA_), rio Tapirapé, Pará, Brazil. *Pariosternarchus amazonensis*: MZUSP 58258 (one, 109.4 mm *L*_EA_), rio Negro, Amazonas, Brazil; MZUSP 48854 (one, 32.9 mm *H*_L_), rio Solimões, Amazonas, Brazil. *Porotergus gimbeli*: MZUSP 83300 (one of 10, 148.8 mm *L*_EA_), rio Amazonas, Amazonas, Brazil. *Sternarchogiton preto*: MPEG 22758 (two of five, 248.2–268.5 mm *L*_EA_), rio Caripetuba, rio Amazonas, Pará, Brazil; MPEG 22737 (one, 19.4 mm *H*_L_), rio Amazonas, Brazil. *Sternarchogiton porcinum*: MZUSP 56319, (one of two, 202.2 mm *L*_EA_), rio Negro, Amazonas, Brazil. *Sternarchogiton* sp.: MZUSP 23428 (one, cs, 8.75 mm *L*_EA_), Lago Tomé, Amazonas, Brazil. *Sternarchella duccius*: MZUSP 57370, (one of two, 146.9 mm *L*_EA_), rio Amazonas, Amazonas, Brazil. *Sternarchella raptor*: USNM 374014, one de five, 71.9 mm *L*_EA_), rio Amazonas, Amazonas, Brazil. *Sternarchella terminalis*: MPEG 3481, (two of five, 154.05–155.3 mm *T*_L_ [regenerated]), Estuário Amazônico, Ilha do Marajó, Pará, Brazil; MZUSP 58187 (one, cs, 160 mm *L*_EA_), rio Amazonas, Pará, Brazil. “*Sternarchella*” *curvioperculata*: MZUSP 23099 (one of four, 183.9 mm *L*_EA_), rio Paraná, São Paulo, Brazil. *Sternarchorhynchus goeldii*: MPEG 1193, (one of four, 148.3 mm *L*_EA_), rio Negro, Amazonas, Brazil; MZUSP 55851, (one, cs, 275 mm *L*_EA_), rio Juapiris, Amazonas, Brazil. Gymnotidae: *Gymnotus* gr. *carapo*: MPEG 3012 (one of four, 232.2 mm *L*_EA_), rio Turiaçu, Maranhão, Brazil; *Gymnotus* gr. *pantherinus*: MZUSP 113616 (one of four, 151.3 mm *L*_EA_), rio Cubatão, São Paulo, Brazil. *Gymnotus cylindricus*: USNM 134701 (one of 23, 178.5 mm *L*_EA_), río Hondo, río Motagua basin, Guatemala. Hypopomidae: *Brachyhypopomus beebei*: MZUSP 103275 (one of nine, 74.6 mm *L*_EA_), rio Jari, Amapá, Brazil. *Hypopomus artedi*: USNM 408442 (one of two, 202. 7 mm *L*_EA_), Paloemeu River, Suriname. Rhamphichthyidae: *Gymnorhamphichthys rosemariae*: MZUSP 56317 (one of two, 116.3 mm *L*_EA_), rio Negro, Amazonas, Brazil. *Hypopygus lepturus*: MPEG 10169 (one of nine, 61.0 mm *L*_EA_), rio Amazonas, Pará, Brazil. *Rhamphichthys marmoratus*: MPEG 8833 (one, 65.8 mm HL; damaged), rio Amazonas, rio Amazonas, Pará, Brazil. Sternopygidae: *Archolaemus orientalis*: MPEG 21509 (one, paratype, 110 mm *L*_EA_), rio São Francisco, Minas Gerais, Brazil. *Distocyclus conirostris*: MZUSP 23316 (one of three, 242.2 mm *L*_EA_), rio Solimões, rio Solimões, Amazonas, Brazil. *Distocyclus guchereauae*: MNHN 2003–0013, 1cs, 222.0 mm *T*_L_, paratypes, Maroni drainage, French Guiana. MNHN 2003–0014, 1, 232.0 mm *T*_L_, paratypes, Maroni drainage, French Guiana. MNHN 2003–0018, 2, 322.0–321.0 mm *T*_L_, paratypes, Maroni drainage, French Guiana. *Eigenmannia humboldtii*: FIELD 56812 (one of seven, 186.2 mm *L*_EA_), Puerto del Rico, Colombia. *Eigenmannia limbata*: MZUSP 75569 (one of two, 160.0 mm *L*_EA_), Lago da Terra Preta, rio Negro, Amazonas, Brazil. *Eigenmannia macrops*: USNM 405266 (one of 16, 103.2 mm *L*_EA_), Cuyuni River, Guyana. *Eigenmannia meeki*: MZUSP 119018 (one of two, paratype), 160.2 mm *L*_EA_, río Pucuro, Panamá. *Eigenmannia trilineata*: MZUSP 111146 (one, 305.0 mm *L*_EA_), rio de La Plata, Argentina. *Japigny kirschbaum*: FMNH 50185 (16, 3 cs, 137.2 mm *L*_EA_), Itabu Creek, New River Drainage, Guyana. MNHN 2008–1201, 110.9 mm *T*_L_, holotype, Mana River, French Guiana. MNHN 2000–5954, 2, 99–111 mm *T*_L_, paratypes, Maroni drainage, French Guiana. *Rhabdolichops troscheli*: MZUSP 57704 (two of 79, 122.2–140.2 mm *L*_EA_), rio Negro, rio Negro, Amazonas, Brazil. *Rhabdolichops zareti*: CAS 57444 (one of 37, 88.9 mm *L*_EA_), río Orinoco, La Providencia, Venezuela. *Sternopygus astrabes*: MZUSP 88795 (one of two, 151. 0 mm *L*_EA_), rio Preto da Eva, rio Negro, Amazonas, Brazil. *Sternopygus macrurus*: MZUSP 32215 (one of 13, 212.6 mm *L*_EA_), rio Amapá, rio Araguari, Amapá, Brazil. MPEG 22756 (two of five, 240.4–245.8 mm *L*_EA_), rio Japurá, rio Solimões, Amazonas, Brazil.

## Results

*Tembeassu* Triques

(Figs 1–10)

**Fig 1:**
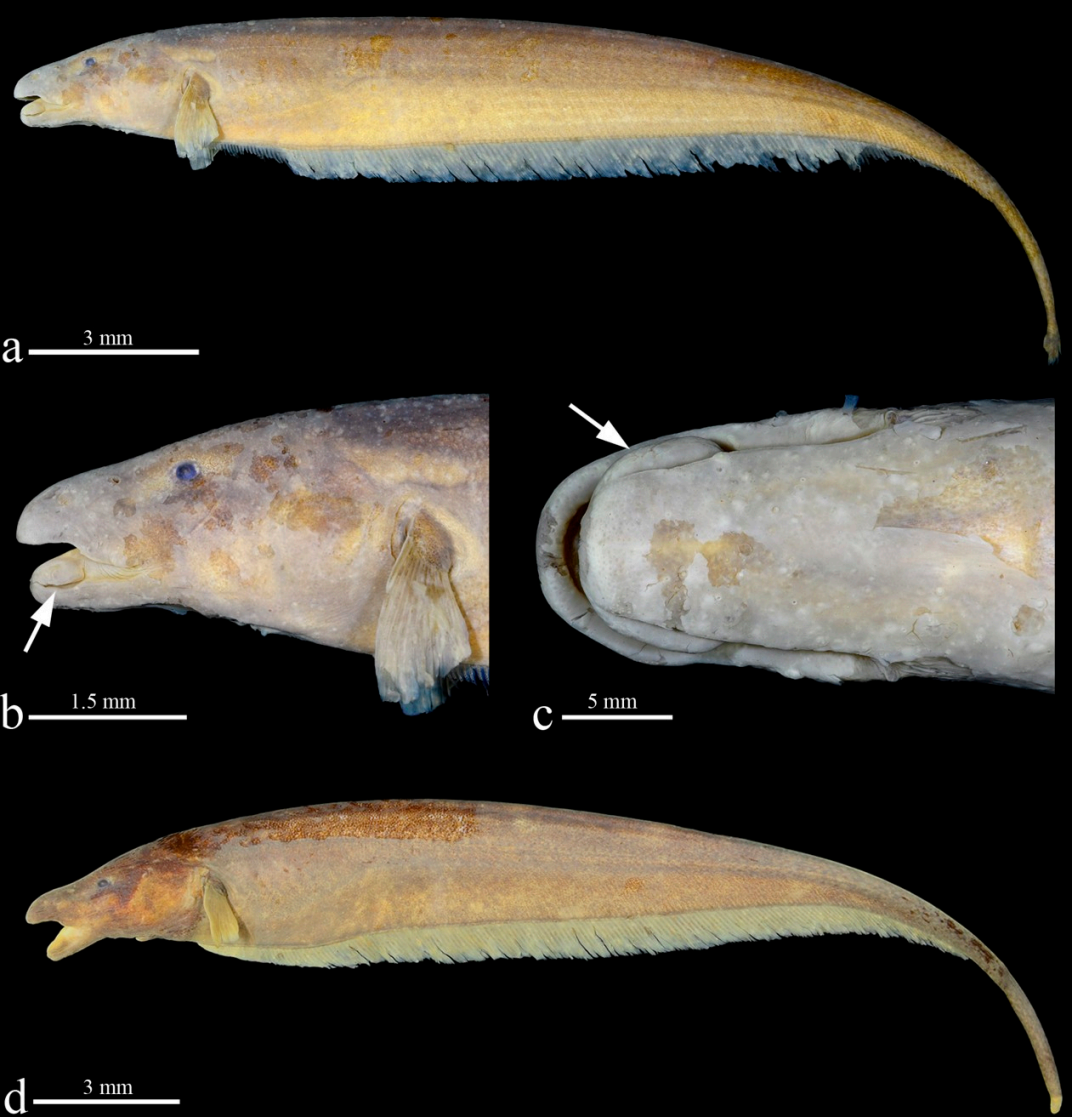
Holotype of *Tembeassu marauna*, male, MZUSP 48510, 188.3 mm *L*_EA_. (A) Lateral view. (B) and (C) head in detail. (D) Paratype, MZUSP 23090, female, 180.7 mm *L*_EA_.

**Fig 2:**
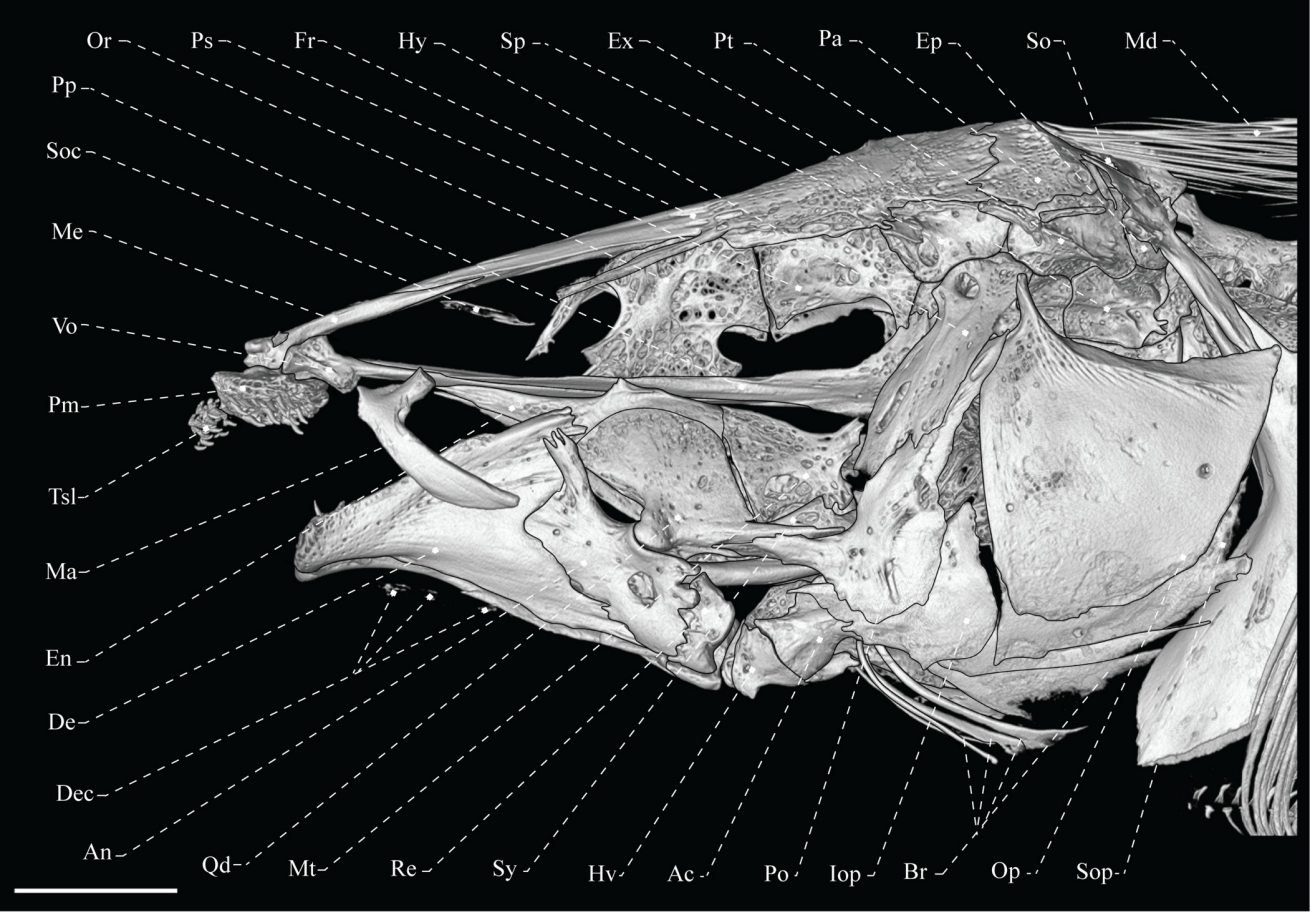
Lateral view of skull of *Tembeassu marauna*, male, holotype, MZUSP 48510, 188.3 mm *L*_EA_.

**Fig 3:**
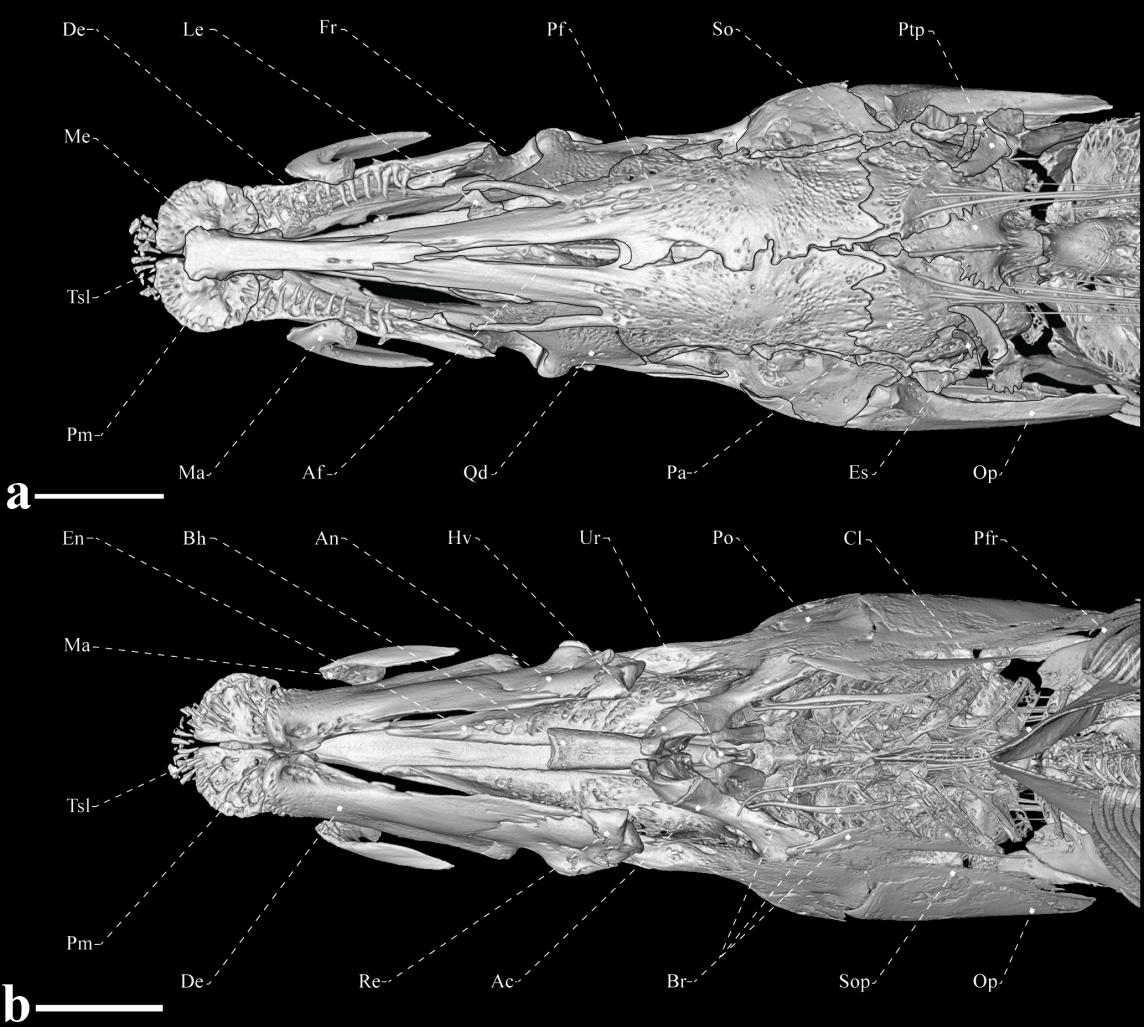
Skull of *Tembeassu marauna*, male, holotype, MZUSP 48510, 188.3 mm *L*_EA_. (A) Dorsal view. (B) Ventral view.

**Fig 4:**
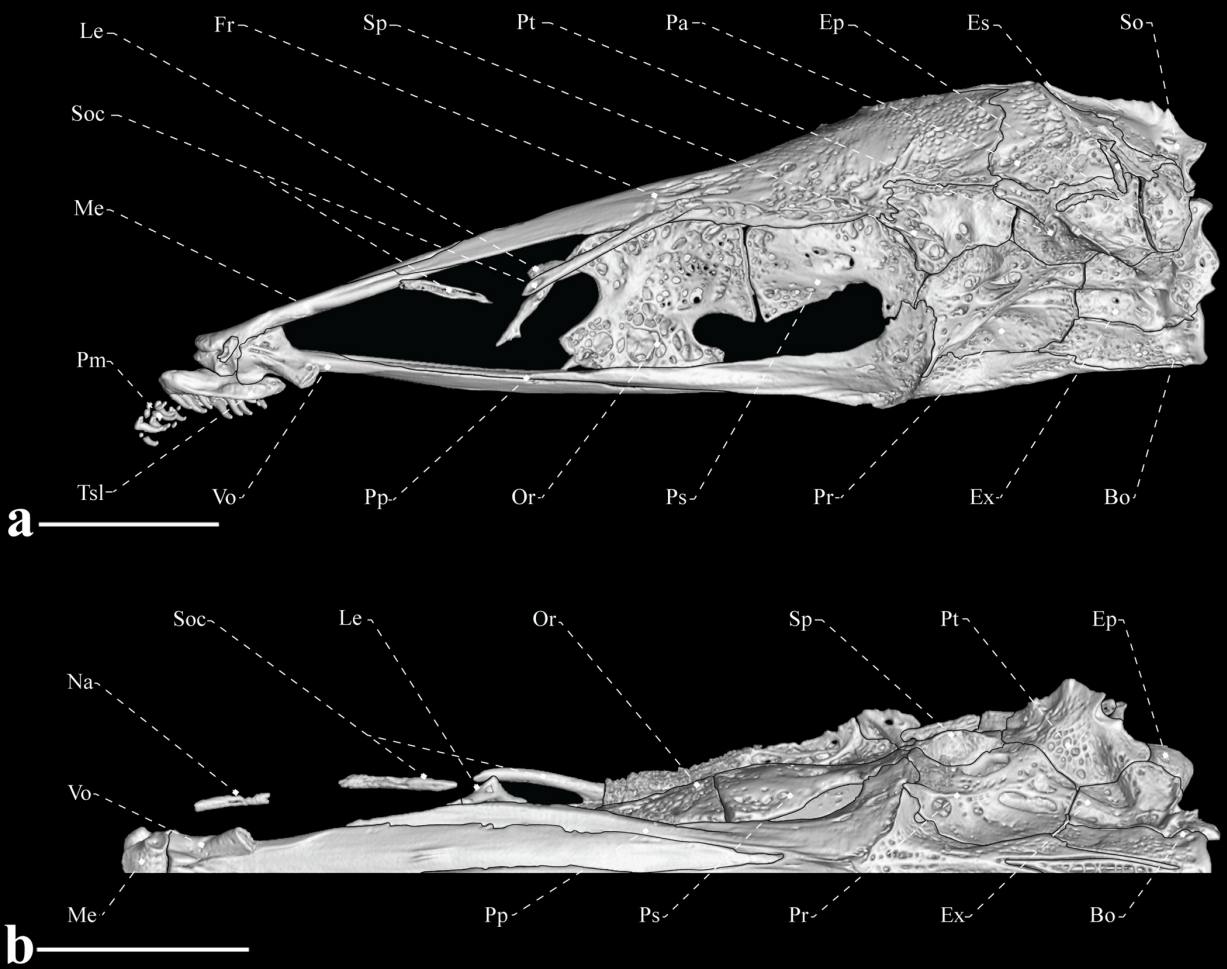
Neurocranium of *Tembeassu marauna*, male, holotype, MZUSP 48510, 188.3 mm *L*_EA_. (A) Lateral view. (B) Ventral view.

**Fig 5:**
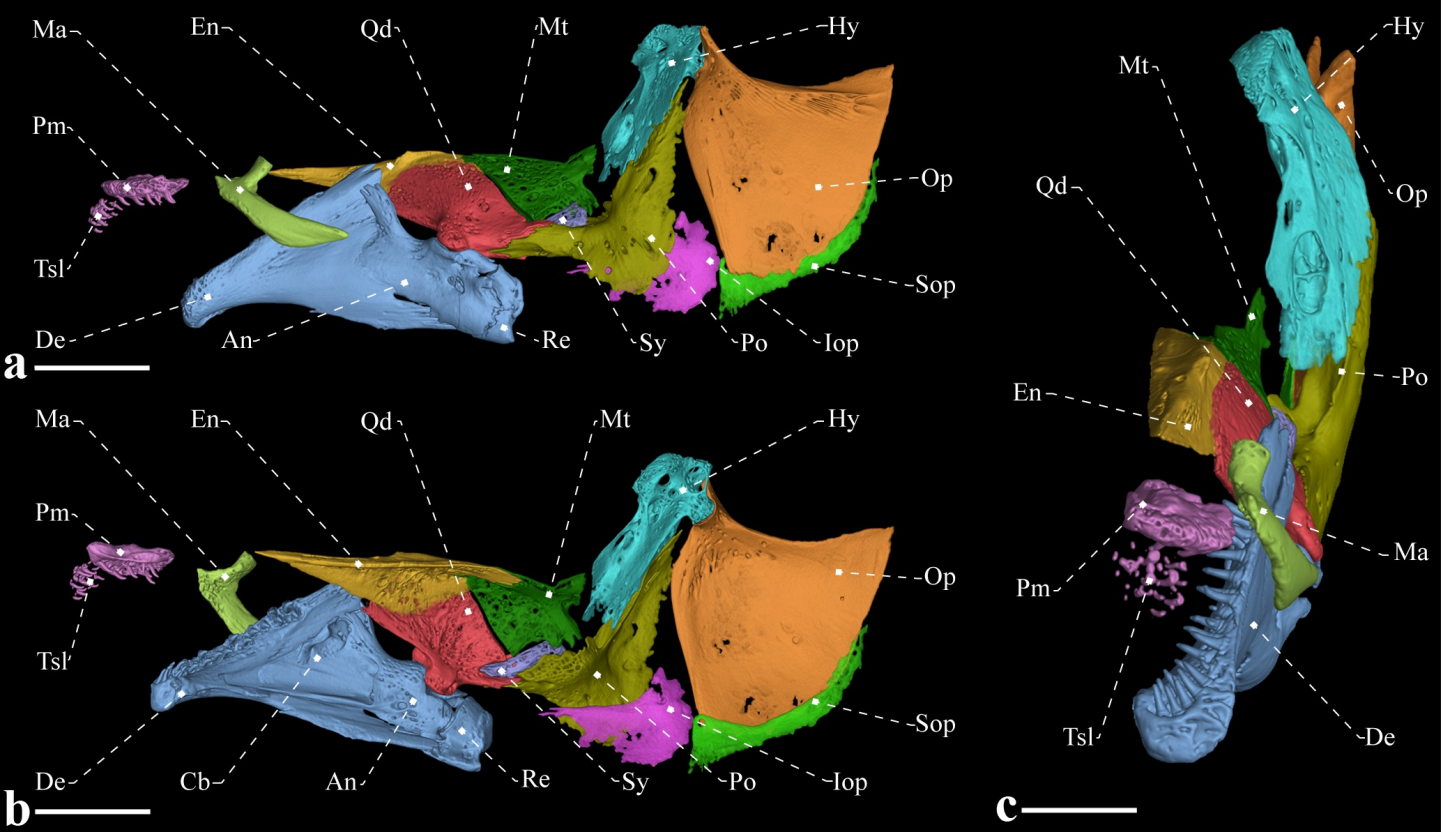
Suspensorium, opercular series and oral jaws of *Tembeassu marauna*, holotype, MZUSP 48510, 188.3 mm *L*_EA_. (A) Lateral view. (B) Medial view. (C) Frontal view.

**Fig 6:**
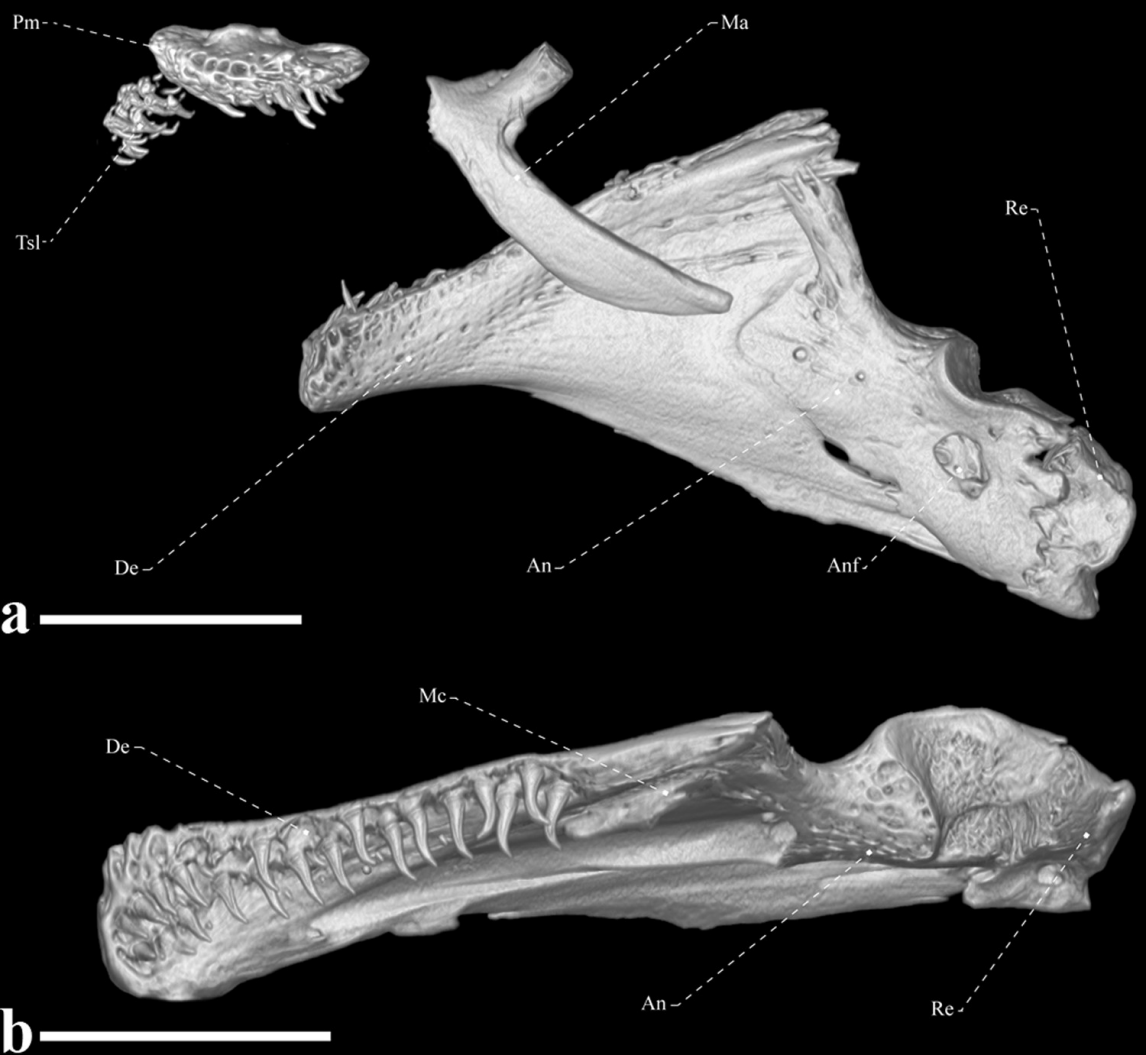
Oral jaws of *Tembeassu marauna*, male, holotype, MZUSP 48510, 188.3 mm *L*_EA_. (A) Lateral view. (B) dorsal view.

**Fig 7:**
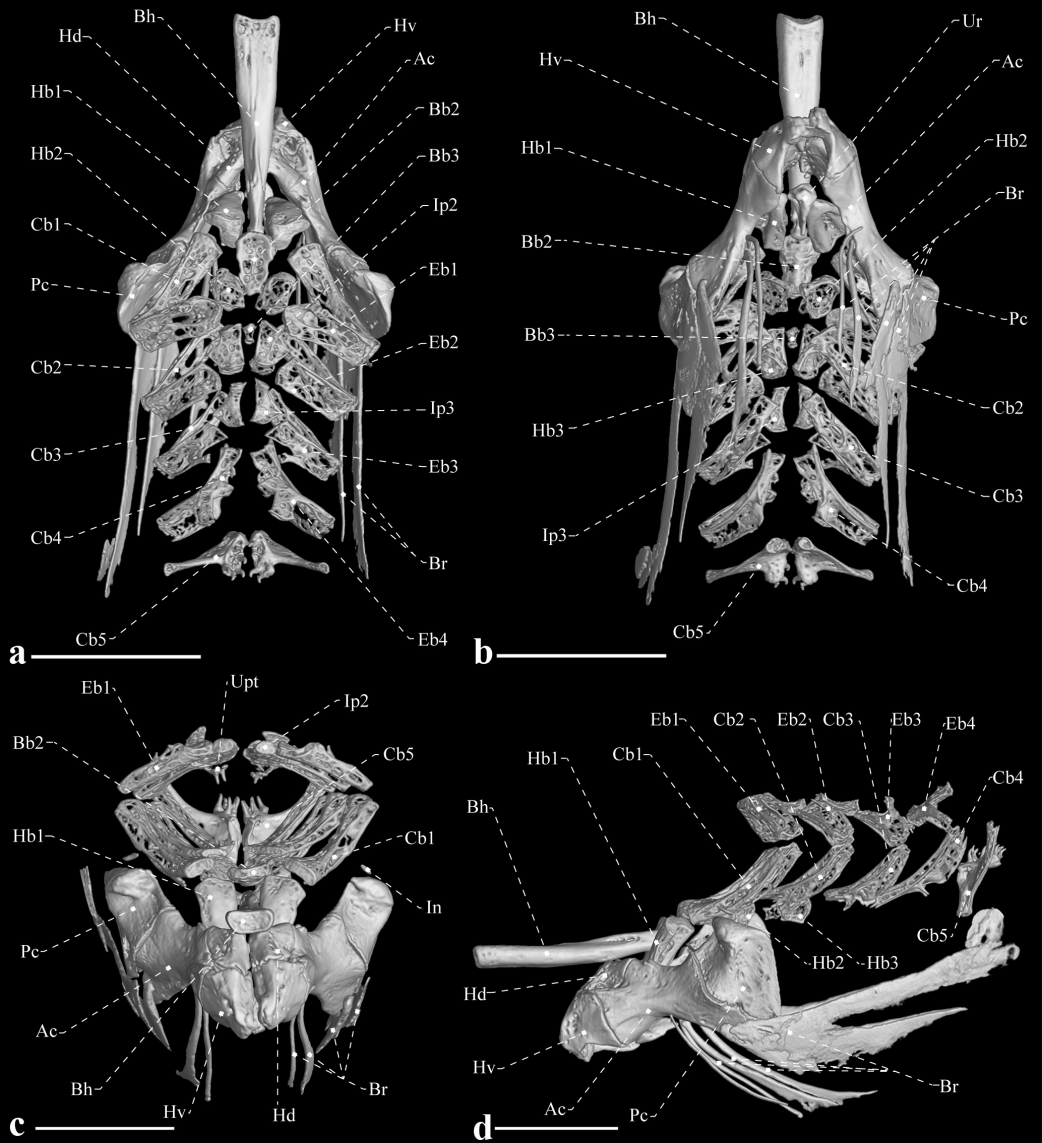
Hyoid bar, branchiostegals and gill arches of *Tembeassu marauna*, holotype, MZUSP 48510, 188.3 mm *L*_EA_. (A) Dorsal view. (B) Ventral view. (C) Frontal view. (D) Lateral view.

**Fig 8:**
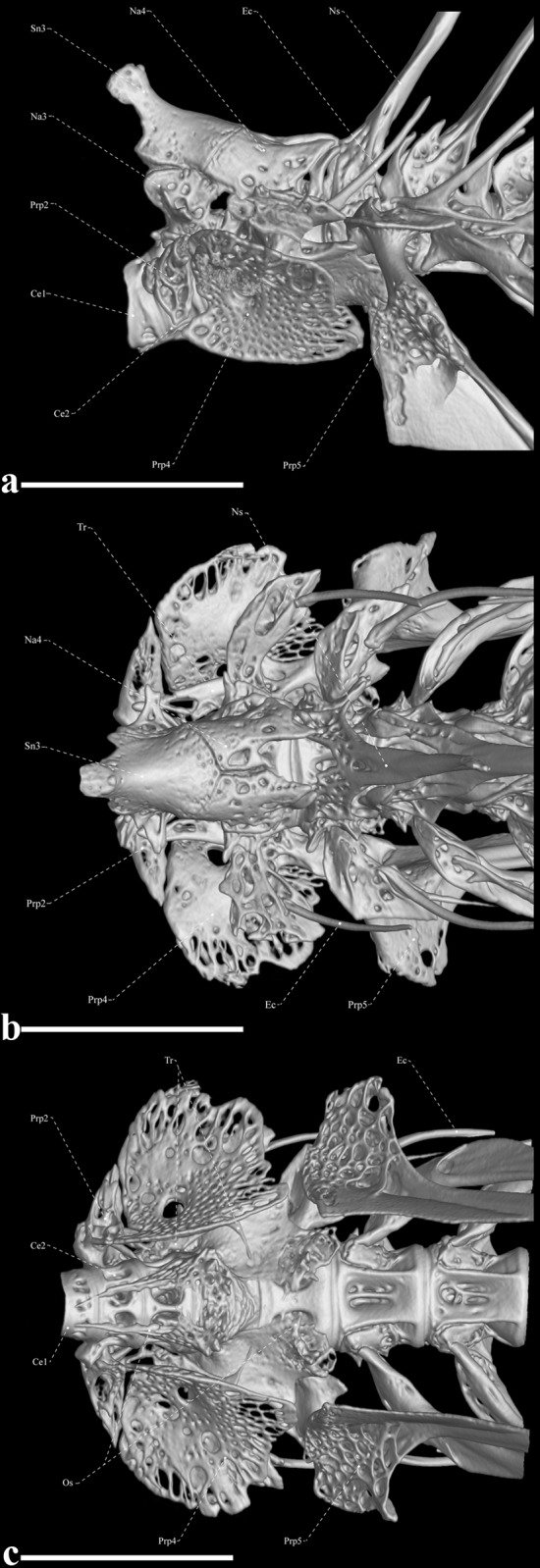
Weberian apparatus of *Tembeassu marauna*, male, holotype, MZUSP 48510, 188.3 mm *L*_EA_. (A) Lateral view. (B) Dorsal view. (C) Ventral view.

**Fig 9:**
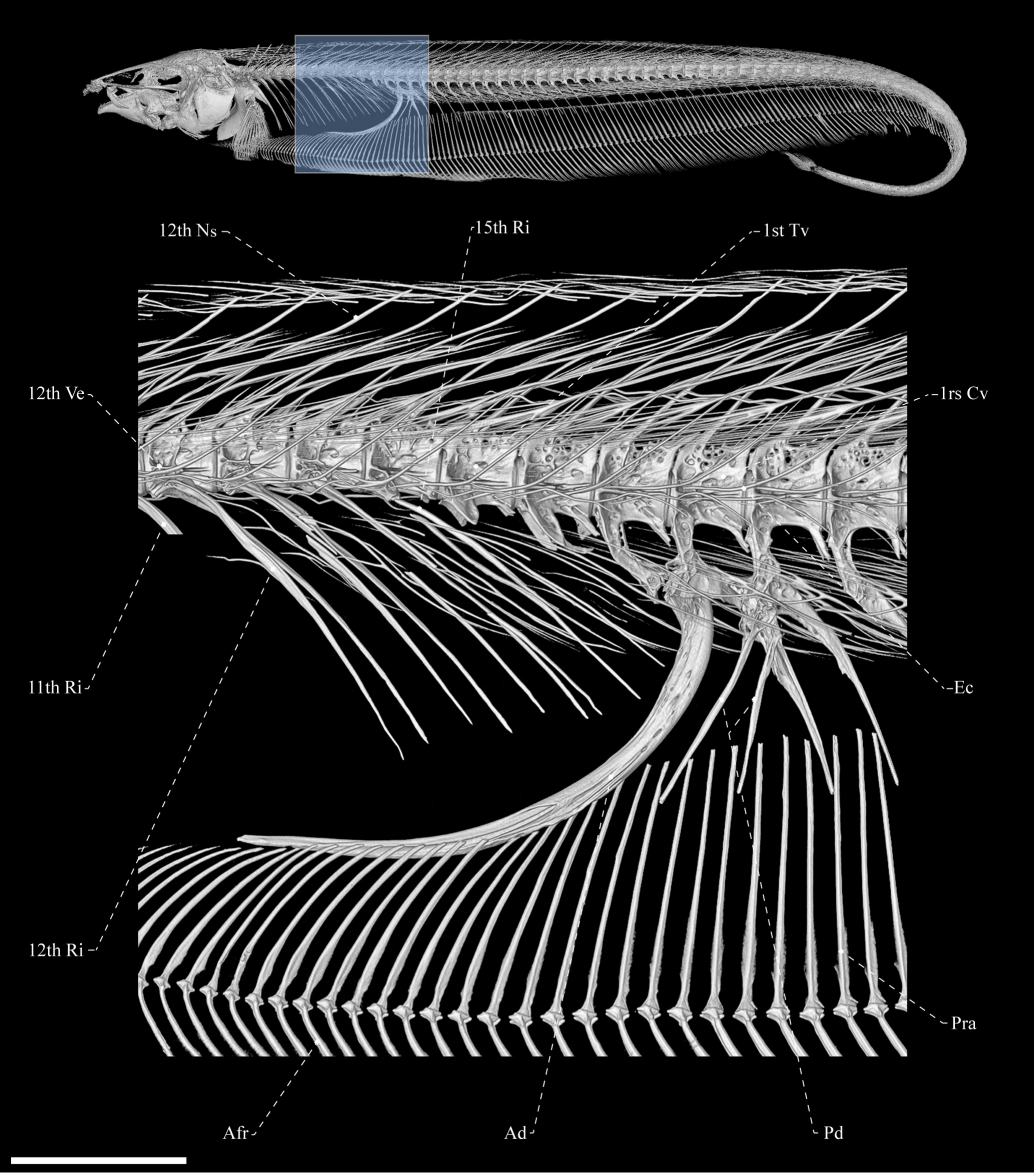
Body (upper) and abdominal cavity (below) of *Tembeassu marauna*, holotype, MZUSP 48510, 188.3 mm *L*_EA_.

**Fig 10:**
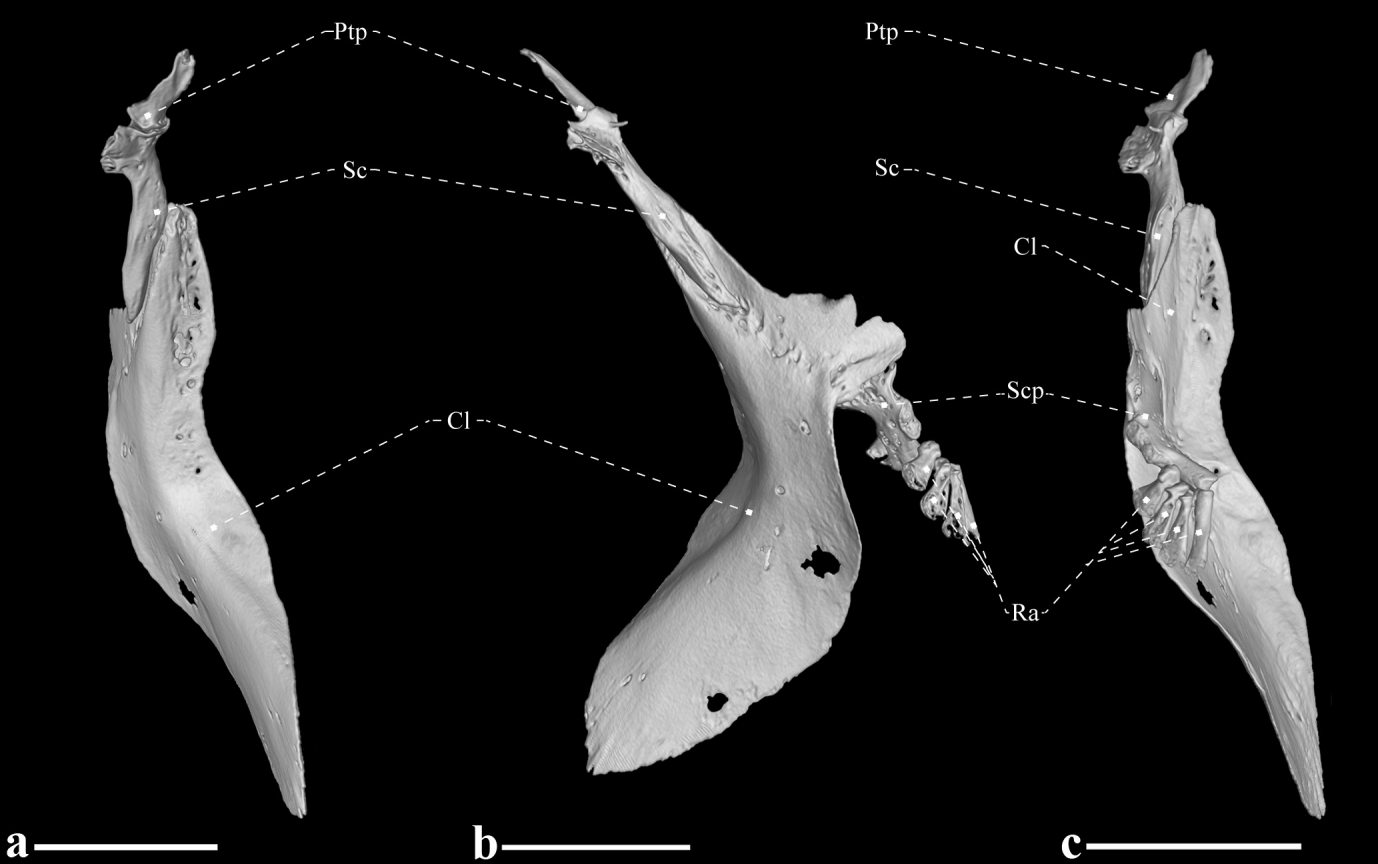
Left pectoral girdle of *Tembeassu marauna*, male, holotype, MZUSP 48510, 188.3 mm *L*_EA_. (A) Frontal view. (B) Lateral view. (C) Posterior view. Mesocoracoid, coracoid and postcleithrum not shown.

*Tembeassu marauna*, Triques [25]: 5 [type species: *Tembeassu marauna*, Triques [25]; type by original designation; masculine].–Campos-da-Paz [28]: 395.–Triques [27]: 121 [listed as examined material].–Albert & Crampton [23]: 83 [cited].–Ferraris *et al*. [30]:15 [checklist].–Albert *et al*. [45]: 46 [cited].

### Amended diagnosis

The monotypic *Tembeassu* is distinguished from all other Gymnotiformes by three autapomorphies: the presence of a rostral patch of extra teeth on the region of the upper lip anterior to the premaxilla (*versus* absence; Figs 1–6; Campos-da-Paz [28], Figs 2–5); a prominent anterior fleshy expansions in both upper and lower lips, with length similar to the premaxillary length (*versus* anterior lip extensions absent or small, with length much smaller than the premaxillary length; Fig 1B and 1C; Campos-da-Paz [28] Figs 3–4); and a fleshy lateral lobe on the lower jaw (= “chin” lobe) hypertrophied (lobe length 14.1–17.4% *H*_L_, lobe width 5.8–8.0% *H*_L_, and lobe depth 6.4–7.4% *H*_L_), oval-shaped, and visible in a ventral view (Fig 1C; *versus* lobe absent, or present, but with a small size [lobe length 2.4–8.7% *H*_L_, lobe width 2.7–4.9% *H*_L_, and lobe depth 2.2–5.1% *H*_L_], fusiform or teardrop-like, and not visible in a ventral view in *Apteronotus sensu lato* [4], *Megadontognathus* Mago-Leccia, *Melanosternarchus* Bernt, Crampton, Orfinger & Albert, *Pariosternarchus* Albert & Crampton, *Sternarchogiton* Eigenmann, and *Sternarchella*.

*Tembeassu marauna* Triques

*Tembeassu marauna*, Triques [25]: 5 [original description: Brazil, Ilha Solteira, Rio Paraná, Mato Grosso do Sul State].–Campos-da-Paz [38]: 1043 [cited].–Campos-da-Paz [28]: 395 [descriptions of osteological features based on type-material; note on conservation status].–Albert & Crampton [46]: 268 [cited].–Triques [27]: 121 [listed as examined material].–Albert & Crampton [23]: 83 [cited].–Crampton [47]: 290 [Table 11.2].–de Santana & Vari [48]: 243 [listed as examined material].–Crampton [49]: 178 [Table 10.2].–Triques [50]: 303 [cited].–Reis *et al*. [51]: 25 [cited].–Ferraris *et al*. [30]:15 [checklist].–Bernt *et al*. [32]: 475 [listed as examined material].

*Apteronotus marauna* (Triques [25]), Albert [4]: 110 [com. nov.; listed as examined material].–Albert in Reis *et al*. [26]: 499 [checklist].–Crampton & Ribeiro [52]: 256–257 [listed; mentioned as having been transferred to *Tembeassu* Triques].

### Diagnosis

As for the genus.

### External anatomy

Morphometric data for examined specimens in Table 1. Body elongate, distinctly compressed laterally. Greatest body depth at vertical through distal margin of pectoral fin. Dorsal profile of body slightly convex to straight. Ventral profile of body slightly convex. Tail compressed and short, ending in small, lanceolate caudal fin. Origin of dorsal electroreceptive filament located on posterior third of body, inserted into narrow mid-dorsal groove extending about 70 scales (filament of holotype and female paratype hardly discernible).

**Table 1:** Morphometrics for types of *Tembeassu marauna* (N = 3). Min = Minimum; Max = Maximum; SD = Standard deviation.

	Holotype	Min	Max	Mean	SD
**Total lenght (mm)**	220.5	220.5	227.1	-	-
**Lenght to end of anal fin (mm)**	188.3	180.7	188.3	-	-
**Head lenght (mm)**	33.2	33.2	33.8	-	-
	**% *L***_**EA**_				
**Head lenght**	17.6	17.6	18.7	18.1	0.6
**Preanal distance**	16.6	16.6	17.7	17.1	0.5
**Prepectoral distance**	17.7	17.7	19.5	18.8	0.9
**Snout to anus**	11.1	11.1	11.7	11.5	0.4
**Body depth at pectoral fin**	13.0	13.0	14.9	14.1	1.0
**Body depth at anal fin**	11.1	11.1	11.8	11.5	0.4
**Body width**	4.7	4.7	6.2	5.5	0.7
**Anal-fin lenght**	82.5	82.4	83.2	82.7	0.4
**Pectoral-fin lenght**	7.7	7.7	8.3	8.0	0.3
**Tail lenght**	17.1	17.1	20.6	18.8	2.5
	**% *H***_**L**_				
**Snout lenght**	43.4	42.5	45.0	43.7	1.3
**Internasal distance**	11.0	11.0	12.5	11.8	0.8
**Snout to posterior naris distance**	26.4	26.4	30.8	27.9	2.5
**Posterior naris to orbit distance**	12.6	10.4	12.8	11.9	1.3
**Internarial width**	13.1	10.6	13.1	11.9	1.8
**Orbital diameter**	5.9	5.9	7.1	6.6	0.6
**Postorbital distance**	51.4	51.4	52.1	51.7	0.4
**Opercular opening**	18.1	16.6	18.1	17.4	0.8
**Suborbital depth**	28.4	26.0	29.5	27.9	1.8
**Interorbital distance**	15.3	14.7	15.9	15.3	0.6
**Head width at opercle**	35.2	35.2	37.6	36.4	1.2
**Head width at orbits**	26.8	26.8	28.1	27.2	0.8
**Head depth at supraoccipital**	54.6	50.9	60.2	55.2	4.7
**Head depth at orbits**	41.8	39.5	41.8	40.6	1.2
**Maxilla lenght**	36.8	36.8	37.8	37.1	0.5
**Oral width**	16.0	15.8	16.8	16.2	0.5
**Lower jaw lobe lenght**	15.2	14.1	17.4	15.6	1.7
**Lower jaw lobe width**	5.8	5.8	8.0	7.0	1.1
**Lower jaw lobe depth**	6.9	6.4	7.4	6.9	0.5
	**% Tail Lenght**				
**Caudal filament depth**	10.5	10.5	10.6	10.5	0.1

Head compressed, greatest width in opercular region and greatest depth in region near parietals. In lateral view, dorsal profile of head slightly convex from upper lip to posterior edge of posterior nostril, moderately ascending and convex from that point to posterior margin of opercle; ventral profile of head concave between lower lip and posterior edge of the fleshy lateral lobe of chin, straight form that point to posterior rim of eye and slightly concave from that point to branchial opening. Snout rounded in lateral view. Mouth terminal, with rictus extending posteriorly to vertical through border of posterior nostril, equal to its width. Upper jaw slightly overlapping anterior margin of lower jaw laterally. Fleshy lateral lobe of lower jaw enlarged, located at anterior half of lower jaw and extending until a vertical through beyond posterior edge of anterior nostril.

Anterior nares tube-like, closer to snout tip than to anterior margin of eye. Posterior nares rounded, with elevated edge, without tube; posterior edge near midpoint between anterior naris and anterior margin of eye. Eyes small, circular, completely covered by thin membrane, on anterior one-half of head length and dorsolaterally oriented. Gill openings limited to posterior margin of opercle and extending above pectoral-fin base. Branchial membranes joined at isthmus. Anus and urogenital papilla adjacent.

First perforated scale of lateral line located above pectoral-fin origin. Scales cycloid, extending from posterior of head to tip of caudal filament. Scales present on mid-dorsal region of body. Scales above lateral line at midbody 10* (1), 11 (1) or 12 (1). Lateral-line scales to vertical through anal-fin terminus 103 (1), 106* (1) or 108 (1).

Pectoral-fin rays ii,13* (1) or ii,14 (2). Distal pectoral-fin margin straight, originating beyond anal-fin origin, at vertical through 9^th^ to 12^th^ anal-fin ray; fourth to seventh pectoral-fin ray longest. Total anal-fin rays 169 [xviii,151] (1), 171 [xxix,142] (1) or 172* [anterior anal-fin rays damaged]. Anal-fin origin at vertical through posterior limit of opercle. Distal margin of anal fin straight. First unbranched rays tiny; rays progressively increasing in size to first branched rays. Branched rays nearly equal length, except for posterior most rays that progressively decrease in size. Caudal-fin rays, 18* (1) or 20 (1) [one specimen damaged].

### Color in alcohol.

Body and head yellow* (2) to dark brown (1), darker at mid-dorsal region of trunk. Origin of pectoral- and anal-fin rays dark brown with interradial membranes translucent. Caudal fin pale dark brown at its base and central portion, translucent posteriorly.

### Osteology

#### Neurocranium

The cranial roof bones are mostly smooth, without ornamentations (Figs 2–4). The paired frontals are the largest bones of the skull roof, occupying about 70% of the skull length. The frontals completely surround the cranial fontanels, which are separated from each other by an exposed epiphyseal bar. The anterior fontanel is approximately three times longer than the posterior one (Fig 3A). The parietal contacts the frontal anteriorly, the supraoccipital posteriorly, and the pterotic laterally. A posterior depression in the parietal presumably serves as attachment site for the epaxial musculature (Figs 3 and 4). The occipital region is composed by median supraoccipital and basioccipital, paired exoccipitals and epioccipitals. The supraoccipital has a sagittal ridge that presumably serve as anchoring site for the deeper trunk muscles. The bone contacts the parietal anteriorly and the epioccipital posteroventrally. The epioccipital is the smallest bone of the occipital region, approximately a half of the pterotic length, and contacts the supraoccipital dorsomedially, the exoccipital ventrally, and the pterotic anteriorly. The exoccipital is a slightly rectangular bone that forms the posteriormost portion of the braincase. That ossification articulates with the basioccipital ventrally, the pterotic anterodorsally, and the epioccipital posterodorsally. The basioccipital contacts the exoccipital dorsolaterally, the prootics anterodorsal and the parasphenoid anteriorly. Internally, basioccipital and exoccipital form a pair of chambers presumably for asteriscus otoliths (Fig 4).

The otic and orbital regions of the neurocranium include the median orbitosphenoid, frontal and parasphenoid, and the paired pterotics, pterosphenoids, sphenotics, and prootics. The pterotic forms the lateral portions of the skull roof and articulates with the prootic anteroventrally, the exoccipital posteroventrally, the epioccipital posteriorly, the parietal and frontal dorsally and the sphenotic anterodorsally. The pterotic exhibits a laterally expanded oblique crest that presumably serves as an anchoring site for the origin of the *levator operculi* muscle (Figs 2–4).

The orbitosphenoid is the anteriormost bone of the orbital region, and contacts the frontal dorsally and the parasphenoid ventrally. The anterior and posterior margins of the orbitosphenoid exhibit a concavity, resulting in a T-shaped pattern. The bone is apparently separate from the pterosphenoid by a segment of cartilage, as the two ossifications are not fully in contact. The pterosphenoid is roughly rectangular and articulates with the parasphenoid ventrally, the prootic posteroventrally and the frontal dorsally. The parasphenoid is an elongate bone contacting the vomer anteriorly and the basioccipital posteriorly. The prootic and pterosphenoid are connected to the parasphenoid posteriorly through its conspicuous ascending ramus (Fig 4).

The sphenotic is a rectangular bone with an anterior projection at its suture with the pterosphenoid. Such a projection apparently serves as origin site for the *dilatator operculi*. At the region near to the suture with the prootic, the sphenotic exhibit a small projection for the articulation with the hyomandibula. The prootic is the largest element of the otic region and, along with the sphenotic, form the articular facet for the hyomandibula. The prootic is pierced by several foramina, including the larger foramen at its dorsal portion for the *truncus hyomandibularis* (Fig 4).

The ethmoid region is relatively elongate and supports the paired lateral ethmoids and the median mesethmoid, and vomer. The lateral ethmoid is relatively small, equal to two thirds of the orbitosphenoid in depth, and located near to the anterior margin of the orbitosphenoid. The bone is axe-shaped and bears a posteromedial expansion towards its median axis (Figs 2–4). The mesethmoid is an elongate bone, about a half of the frontal length, with a small anterior projection that articulates with the premaxilla. The vomer is located ventrally to the anterior portion of the parasphenoid and exhibits a small anterolateral projection on each side.

#### Autogenous ossifications of the laterosensory system

Small tubular ossifications strictly associated with the cephalic laterosensory canals are poorly detected by the methodology performed herein. Thus, other minute ossifications may be present in addition to the following described elements. The anteriormost ossification of the supraorbital sensory canal is a mid-sized element, with length equivalent to that of the lateral ethmoid, and located anteriorly to this bone (Fig 4). The anterior element is followed by a larger ossification that is fused to the frontals at its posteriormost portion (Figs 3 and 4). There are two elongated ossifications partially corresponding to the extrascapular, located at the posterior margin of the parietal and dorsoposterior portion of the sphenotic (Fig 4A). These elements are apparently fused to the parietal and pterotic. Three autogenous ossifications associated with the mandibular canal are detected ventrolaterally to the lower-jaw bones (Fig 2).

#### Suspensorium and oral jaws

The suspensorium consists of the hyomandibula, symplectic, quadrate, metapterygoid, and endopterygoid (Fig 5). An ossified autopalatine was not detected. The endopterygoid is anteriorly narrow, and progressively expanded posteriorly. The ossification is overlaid laterally by both by the quadrate and metapterygoid. The endopterygoid exhibit a small crest with a small process on its intermediate portion, supposedly for the attachment of the pterygocranial ligament. The quadrate is the largest suspensorial bone, fan-shaped, with its posterolateral portion articulating with the preopercle and posteromedial face with a socket for the accommodation of the symplectic. The metapterygoid is located posterior to the endopterygoid and posterodorsally to the quadrate. That bone is approximately trapezoidal in shape, with a small posterodorsal projection.

The main axis of the hyomandibula is slightly oblique, with nearly straight anterodorsal and posterodorsal surfaces for the articulation with the sphenotic and pterotic, respectively. The opercular condyle of the hyomandibula is directed posteroventrally. There are three foramina in the hyomandibula: one at the anterodorsal portion of the bone for the entering of the *truncus hyomandibularis*; a second with roughly in the ventral portion, with roughly twice of the size of the first, for the exit for the *truncus hyomandibularis*; and a third at the posterodorsal portion of the bone, with diameter equivalent to anterodorsal foramen, for the exit for the recurrent ramus of anteroventral part of anterior lateral-line nerve. The symplectic is a rod-like element mostly located on the medial face of the suspensorium and articulating posteriorly with the hyomandibula (presumably via hyosymplectic cartilage), dorsally with the metapterygoid and anteriorly with the quadrate (Fig 5).

The edentulous maxilla is elongated and bears a long posterodorsal process with about two thirds the descending blade of the maxilla. The anteroventral margin of the descending blade is nearly straight with the ventral portion smoothly curved. There are 15, 21* or 22 rostral teeth directly implanted into the soft tissue of the upper lip (Figs 5 and 6). These teeth are conical and with a posteriorly directed curvature. The premaxilla is approximately flattened, rectangular, with rounded borders and a concavity on dorsal surface that serves for articulation with the mesethmoid. Premaxilla with 11 conical teeth arranged in three irregular rows* [rows not discernible in the x-rayed paratypes]. The ossifications of the lower jaw consist of the dentary, angulo-articular, retroarticular, and coronomeckelian bone (Fig 6). The anterodorsal margin of the dentary is slightly straight and its posterior portion its distinctly forked. The ossification bears 22 teeth [around five replacement teeth] in the holotype and around 30 teeth on the remaining types. The dentary teeth are arranged into two rows that extend from the mandibular symphysis to the second third of the bone.

Meckel’s cartilage not detected, but presumably present as a triangular block located anterolateral to the angulo-articular. The coronomeckelian bone is located at the middle of the meckelian fossa, between the angulo-articular and dentary. The angulo-articular is triangular-shaped, with a dorsal process oriented anteriorly and passing lateral to the posteriormost portion of the dentary. Only the angulo-articular contributes to the socket of the articular facet with the quadrate. The retroarticular is the posteriormost bone of the lower jaw, approximately trapezoidal in shape, and does not contact the quadrate (Figs 5 and 6).

#### Opercular series

The opercular series is composed by the preopercle, opercle, subopercle, and interopercle (Fig 5). The preopercle is L-shaped, with an anteromedial fossa for the origin of the *segmentum facialis* of the *adductor mandibulae*. The dorsalmost portion of the bone lies medial to the posterior margin of the hyomandibula. The opercle is approximately triangular in shape, with a concave dorsomedial margin that presumably received the fibers of the *levator operculi* muscle. Its anterodorsal process passes lateral to the posteriormost margin of the hyomandibula and presumably serves as insertion site for the *dilatator operculi* muscle. The interopercle is teardrop-shaped, poorly ossified, located medial to the preopercle, and extending from the anterior margin of the preopercle to the anteroventral border of the opercle. The subopercle is an elongated and sickle-shaped bone located ventromedial to the opercle and weakly ossified likewise the subopercle.

#### Gill arches, hyoid bar and branchiostegal series

Only ossified elements are accounted in the following descriptions (Fig 7). Dorsally, each side of the branchial skeleton consists of four ossified epibranchials (1–4), two ossified infrapharyngobranchials (2–3), and an upper pharyngeal toothplate. Epibranchials 1 and 2 are similar in shape, exhibiting a flattened rectangular pattern. Epibranchial 3 is similar to epibranchials 1 and 2, however, with a tapered prominent process on its posterior border. Epibranchial 4 is forked posteriorly with each prong resulting in a Y-shape. The circular upper pharyngeal toothplate is a small ossification that anchors two small conical teeth, with distal tips recurved.

Ventrally, the branchial skeleton is composed of three hypobranchials (1–3), five ceratobranchials (1–5), and two ossified basibranchials (2–3). Hypobranchial 1 is bullet-shaped, with the anterior tip allocated ventrally to the posterior portion of the basihyal. Hypobranchial 2 is trapezoidal, with length equal to two thirds that of hypobranchial 1. Hypobranchial 3 displays a pentagonal shape, with rounded margins, and roughly half the hypobranchial 1 length. The ceratobranchials are the largest ossified elements, with a rectangular and elongate shape. Ceratobranchials 3 and 4 possess a small lateral process on their anterolateral margin. Ceratobranchial 5 is more arched and oblique than the other ceratobranchials and is expanded to anchor seven (left side) or nine (right side) conical teeth [only for the holotype; not analyzed in the remaining types].

The interhyal is a small and cylindrical ossification located dorsal to the posterior ceratohyal. The latter bone is approximately trapezoidal, with straight posterior and dorsal margins and a distinct notch ventrally. The anterior ceratohyal is ax-shaped and anteriorly contacts the dorsal and ventral hypohyals. The ventral hypohyal is nearly triangular; the dorsal hypohyal is fan-shaped and contacts the posterior third of the basihyal. The basihyal is a cylindrical and elongated bone, with a wider anterior portion wide that gradually tapers toward posterior. This bone possesses a small dorsal crest on its posteriormost portion. The urohyal is notably reduced and drop-shaped. Four branchiostegal rays are present: the two anteriormost articulate with the ventral margin of the anterior ceratohyal and two posteriormost with the posteroventral portion of the same bone. All branchiostegal rays are distinct in shape. The first one is homogeneously slender; the second is similarly slender, but gradually wider posteriorly; and the two posteriormost branchiostegal rays are extremely decalcified and much broader than the remaining branchiostegal rays, assuming an approximately triangular shape.

#### Weberian apparatus and post-cranial axial skeleton

The third and fourth neural arches and supraneural 3 are the largest elements of the Weberian apparatus (Fig 8). Supraneural 3 is ax-shaped (Fig 8A), with an anterodorsal projection with a rounded tip that contacts the supraoccipital and exoccipital. The tripus is narrow anteriorly, wide and laminar in its midsection and tapers again at its posteromedial portion. The first vertebral centrum is compressed, resulting a disk-like pattern, and connected anteriorly to the basioccipital. The second centrum is approximately twice the length of the first centrum and anchors a pair of laterally expanded laminar parapophyses. The anterodorsal margin of the third centrum is associated with both its neural arch and that of the fourth vertebra. The neural arch of the fourth vertebra sutures to supraneural 3 anterodorsally and the third neural arch anteroventrally (Fig 8B). Dorsolaterally, the fourth vertebral centrum supports a dorsal laminar expansion posteriorly directed, similar to the projection of the second parapophysis. The expanded fourth parapophysis is nearly wing-shape in dorsal and ventral views, rectangular in lateral view, with a rounded margin and posteriorly directed. The os suspensorium are paired elements, with the anteriormost triangular and tapering anteriorly until the midpoint of the first centrum, and the posteriormost expanded until the posterior margin of the fifth vertebra (Fig 8C). The scaphium and intercalarium are present, but their morphologies were not clear in our μCT scanned specimen.

There are 18 abdominal vertebrae (two transitional vertebrae) including the Weberian apparatus vertebrae (Fig 9). In the anteriormost post-Weberian apparatus vertebrae, the neural spines emerge from the anterior region of the centrum, but become progressively more posteriorly displaced and more oblique relative to the body axis, especially in the posterior third of the body (Figs 8 and 9). The length of the posteriormost neural arches spans about the two subsequent vertebral centra. Anteriormost (Weberian) vertebrae are similar in shape, except for the fifth vertebra, which is greatly modified. This vertebra exhibits a posteriorly directed dorsal expansion from the lateral surface of the centrum, comparable to the fourth vertebra and its associated rib is, supposedly, fused to the parapophysis that anchors a wide wing-like expansion. The morphological pattern of the intermuscular bones and caudal vertebrae is similar to that described by Hilton *et al*. [36], except for the neural and hemal spines of the caudal vertebrae, which are oblique to the body axis and span about three and two vertebrae, respectively. Associated with the posteroventral wall of the abdominal cavity, there are one sickle-shaped, anteriorly displaced hemal spine and two small and thin posteriorly displaced hemal spines in all specimens.

#### Fins and supports

All anal-fin rays are segmented and branched posterior to ray 20–30 (anal-fin damaged in the holotype); no rays branched more than once. The proximal radials are the only ossified elements supportive of the anal fin detected in the μCT and radiographs, but fully cartilaginous distal radials are likely present. There are two or three proximal radials between each adjacent hemal spines. Except for the first one, all proximal radial have an associated fin ray (Fig 9). Anterior to the tip of the anterior displaced hemal spine there are about 18 proximal radials that are oriented progressively more obliquely so as to become perpendicular to the body axis by about fin ray 25–38. The posteriormost proximal radials are arranged gradually more oblique in the opposite direction than the anteriormost ones.

The elements of the pectoral girdle include the posttemporal, supracleithrum, postcleithrum, cleithrum, scapula, mesocoracoid, and coracoid (Fig 10). The posttemporal is small, partially overlaps the supracleithrum, and lies near to the epioccipital-pterotic suture. The supracleithrum is elongate and overlaps the posterodorsal portion of the cleithrum. The postcleithrum is a laminar, extremely decalcified (hardly detected in the μCT scanned specimen) and shield-shaped element that is loosely connected to the pectoral girdle. The cleithrum is large, L-shaped, with a medial depression, and contacts its antimere anteroventrally. The scapula is a L-shaped element that contacts the mesocoracoid anterodorsally and the coracoid ventrally. The mesocoracoid is thin, poorly ossified in the specimen analyzed, T-shaped and attached to the medial surface of the cleithrum and coracoid. The coracoid is broad dorsally, becoming narrow anteroventrally and tapered toward its anteroventral tip. The only ossified supports for the pectoral fin rays detected herein consist of four pectoral radials.

The caudal skeleton is composed of a single element, with the hypural plate nearly triangular. A single cartilage (not detected by the micro computed tomography, but exposed in one specimen) supports the caudal-fin rays.

### Distribution

*Tembeassu marauna* is currently known only from its type-locality at the Rio Paraná, municipality of Ilha Solteira (at the Ilha Solteira dam area), Mato Grosso do Sul state, Brazil.

## Discussion

### Phylogenetic relationships of *Tembeassu*

**Fig 11:**
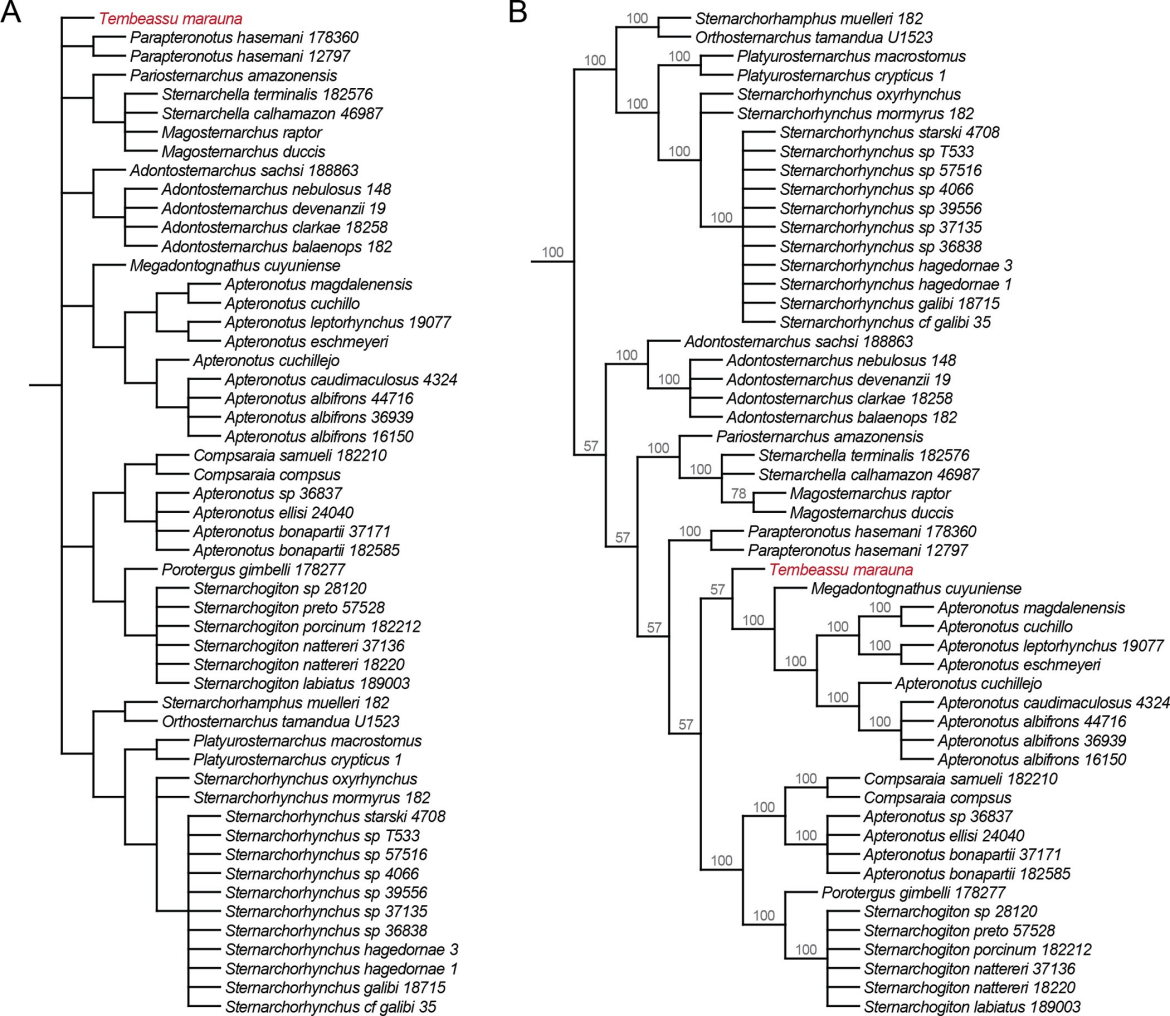
Diagram of interrelationships among the Apteronotidae genera. (A) Strict consensus and (B) majority-rule consensus of 99,999 equally parsimonious trees (586 steps) obtained in the maximum parsimony analysis; numbers represent the percent frequency of each node in the majority consensus [Complete trees provided in Supporting information S2–3].

The μCT scanning technique performed herein disclosed previously inaccessible osteological data from the enigmatic *Tembeassu marauna*. In order to try to infer the phylogenetic position of *Tembeassu*, we tentatively included *T*. *marauna* in a modified version of the morphological matrix presented in Tagliacollo *et al*. [53], and performed a maximum parsimony analysis that recovered 99,999 (overflow) most parsimonious trees, with 586 steps, which were synthesized in the consensuses in Fig 11 (see S2 and S3 Files for the whole consensus and majority-rule tree, respectively). In the following discussion, character numbering follows that of Tagliacollo *et al*. [53].

This analysis unequivocally placed *Tembeassu* within the Apteronotidae, based on six synapomorphies (characters numbered as in Tagliacollo *et al*. [53]): proximal portion of the hyomandibula narrow, with articulating surface facing dorsally (ch. 131); preopercle narrow and curved with the ventral margin of the anterior limb unossified (ch. 136); anterior pharyngobranchial unossified (ch. 143; pharyngobranchial not detected by the μCT); dorsal surface of the basihyal convex along its long axis, forming a ridge (ch. 156); presence of a dorsosagittal electroreceptive fleshy filament (ch. 183); and presence of the caudal fin (ch. 231). Some of proposed synapomorphies for the Apteronotidae involving cartilages and soft tissues (Tagliacollo *et al*. [53]: chs. 91, 92, 119, 143, 149, 221) could not be verified as only mineralized structures were detected by the μCT scan. Conversely, it should be noticed that three putative osteological synapomorphies for the family recovered by Tagliacollo *et al*. ([53]: 36–38) and literature therein, were neither confirmed in our analysis nor found in *Tembeassu* and deserve additional comments, as follow:

The possession of third and fourth infrapharyngobranchials ossified (ch. 144, state 1). In *Tembeassu*, only the second and third infrapharyngobranchial are ossified (Fig 7), a pattern also found in most apteronotid genera examined herein [*Sternarchogiton*, *Sternarchella*, and “*Apteronotus*” *brasiliensis* (Reinhardt)] as well as in previous reports of the literature (*Adontosternarchus*, Mago-Leccia *et al*. [19]: 7, Fig 5; *Megadontognathus*, Campos-da-Paz [38]; *Orthosternarchus*, Hilton *et al*. [36]: 17, Fig 14; *Sternarchorhamphus*, de Santana and Vari [8]). Other non-apteronotid gymnotiforms also possess a cartilaginous fourth infrapharyngobranchial [8]. Within Apteronotidae, only *Sternarchorhynchus* and *Platyurosternarchus* species have the fourth infrapharyngobranchial well-ossified in addition to the second and third that are also ossified. The additional ossification of the fourth infrapharyngobranchial has been consequently interpreted as a synapomorphy for the clade *Sternarchorhynchus* and *Platyurosternarchus* (de Santana and Vari [8]: 252, ch. 62, state 1). Elucidation of the conflicts among the original matrix of Tagliacollo *et al*., the other reports in the literature, and our observations demands a broader osteological review across all gymnotiform genera. Such an achievement lies beyond the scope of the present study and, in order to reduce noise in the present analysis, we provisionally removed character 144 from the matrix.

The posterior surface of the *second* ceratobranchial with a medially oriented process (ch. 151, state 1). Tagliacollo *et al*. [53] credited this character to Albert ([4]: 38, ch. 156, state 1; p. 38) and Triques ([54]: 112, ch. 39). Conversely, Albert [4] referred to a “posterior process of *fourth* ceratobranchial”, also citing Triques [54]. To further complicate the issue, the original character of Triques describes a tapered process on the anterolateral surface of the *fifth* ceratobranchial, and that author considered a synapomorphy for Sternopygidae ([54]: 112, ch. 39). In any case, neither the second nor the fifth ceratobranchial of *Tembeassu* exhibit any process. Only the fourth ceratobranchial has a tapered process but along its anteroventral portion (Fig 7D), thus not matching any of the characters proposed by previous studies. In light of these discrepancies, we also removed character 151 from the matrix, pending an osteological review of the entire Gymnotiformes.

The presence of a single displaced hemal spine (DHS) posterior to a large anterior spine (ch. 182, state 1). Some apteronotids indeed possess this condition (*e*.*g*., *Orthosternarchus*, Hilton *et al*. [36]; *Adontosternarchus*, Albert [4]; *Compsaraia*, Albert & Crampton; *Pariosternarchus*, Albert & Crampton). However, several other apteronotid genera exhibit a varied number of DHS posterior to the large anterior spine, such as: one or two in *Platyurosternarchus crypticus* and *P*. *macrostomus* [48] and two in *Sternarchorhynchus galibi*, *S*. *hagedornae*, *S*. *mormyrus*, and *S*. *oxyrhynchus* [8]. *Tembeassu* possesses two posterior DHS in all analyzed specimens (Fig 9). These corrections were implemented in the matrix and, as a result, the optimization of character 182 is ambiguous at the base of Apteronotidae.

An additional miscoding was detected in character 188 (“number of anal-fin rays”) of Tagliacollo *et al*. [53], since *Sternarchorhynchus mormyrus* and *S*. *oxyrhynchus* were mistakenly assigned with “state 2” (“160–199 rays”), although both species do, in fact, exhibit state 3 (i.e., “200–299 rays”; de Santana & Vari [8]). We assigned the correct character state for these taxa in our matrix prior to the analysis and, as a consequence, state 2 is optimized as a synapomorphy for all the clade clustering all species *Sternarchorhynchus* of the analysis except *S*. *mormyrus* and *S*. *oxyrhynchus*. This has an impact on the optimization of character 188 concerning node 299 (Sternarchorhynchini) of Tagliacollo *et al*. [53]. The same sorts of problems seem to be widespread across the whole original matrix, indicating the critical need of an exhaustive review of the gymnotiform anatomy and previously proposed anatomical characters under a detailed contemporaneous phylogenetic perspective.

The inclusion of *Tembeassu* in the parsimony analysis of the morphological dataset of Tagliacollo *et al*. [53] strongly affects the internal resolution of the Apteronotidae in the resulting strict consensus, which assembles a few minor clades (e.g. *Megadontognathus* + *Apteronotus s*. *s*.; *Platyurosternarchus* + *Sternarchorhynchus*; *Pariosternarchus* + *Sternarchella*) into a large basal polytomy (Fig 11A). *Tembeassu* is placed therein isolated from all remaining apteronotids. Curiously, *Tembeassu* also appears as part of a large polytomic structure within Apteronotidae in a strict consensus tree presented by Triques ([27]: 137). However, in the data matrix made available therein ([27]: 124–125, Table 1) more than 50% of character states referring to *T*. *marauna* were assigned as “uncertainties” (i.e., “?”), which, at least in part, could explain the poor resolution of that tree (moreover, some character states are actually missing for a few other taxa, which precludes any objective test of the results).

**Fig 12:**
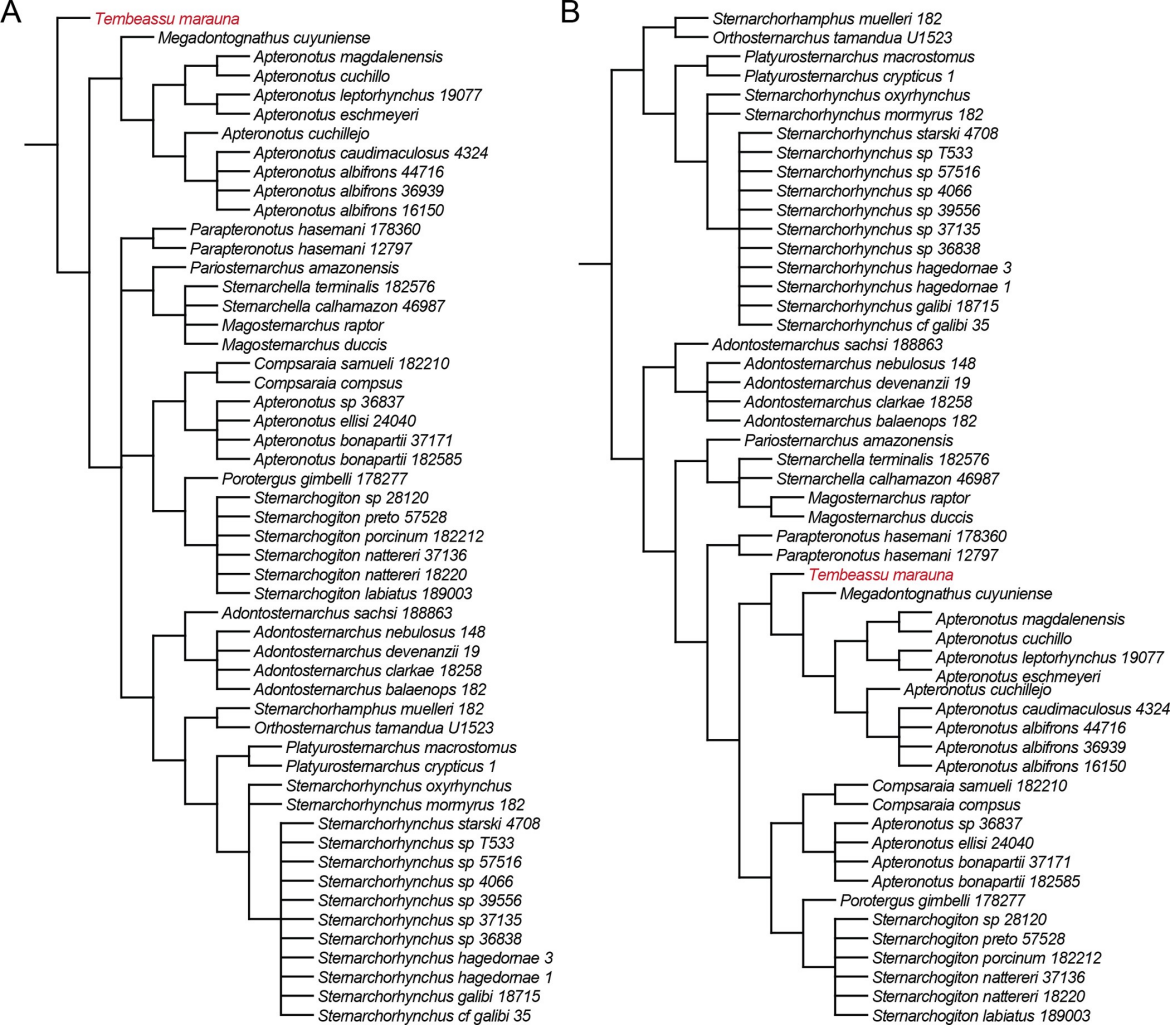
Two alternative phylogenetic placements of *Tembeassu* among the 99,999 sampled MPT’s. (A) as the sister-group to all remaining apteronotids (strict consensus of 42,566 MPT’s; complete tree provided in Supporting information S4). (B) as the sister-group to the clade *Megadontognathus* + *Apteronotus s*. *s*. (strict consensus of 57,433 MPT’s; complete tree provided in Supporting information S5).

Nevertheless, inspection of the 99,999 MPT’s obtained herein reveals that the uncertain position of *Tembeassu* and the large polytomy at the base of Apteronotidae of our strict consensus derives from only two alternative placements of that genus: (1) as the sister group to all remaining apteronotids (Fig 12A; see S4 for the whole tree), or (2) as the sister group to the clade *Megadontognathus* + *Apteronotus s*. *s*. (Fig 12B; see S5 for the whole tree). Under the first scenario, the clade composed by all apteronotid genera except *Tembeassu* is supported by four synapomorphies: fossa in posttemporal region (ch. 88, state 1); dorsal margin of opercle straight (ch. 138, state 1); anterior pharyngobranchial unossified (ch. 143, state 1); and 100–159 anal-fin rays (ch. 188, state 1). In the second scenario, the clade including *Tembeassu*, *Megadontognathus*, and *Apteronotus s*.*s*. is supported by the dermal ossifications of cranial skeleton composed of lamellar or cancellous bone (ch. 90, state 0), and body cavity associated with 16–19 vertebrae (ch. 197, state 0). All optimized synapomorphies in both scenarios are homoplastic across the Gymnotiformes.

The placement of *Tembeassu* as sister to the clade *Megadontognathus* + *Apteronotus s*. *s*. is predominant (57%) among the 99,999 MPT’s obtained from the maximum parsimony analysis performed herein (Fig 11B). In addition, the relationships among apteronotids obtained in this topology is the same recovered when a maximum parsimony analysis of the same dataset is implemented without the inclusion of *Tembeassu*. Thus, despite the necessity of extensive review of the datasets currently available in the literature, a closer relationship among *Tembeassu*, *Megadontognathus*, and *Apteronotus s*.*s*. seems to be the most likely hypothesis to date.

### Taxonomic status of *Tembeassu*

Albert ([4]: 126, Appendix 4) included *Tembeassu marauna* into his *Apteronotus sensu stricto*. This proposition was subsequently rejected by Campos-da-Paz [28] who resurrected the genus and demonstrated that most character states used to define *Apteronotus s*.*s*. are absent in *Tembeassu*. Specifically, Campos-da-Paz [28] was unable to verify in *Tembeassu* the occurrence of one of the diagnostic characters for *Apteronotus s*.*s*., namely, the anterior limb of angulo-articular shorter than its posterior limb (Albert [4]: 21, character 48). This was assumed as uncertain by Campos-da-Paz [28: 399] as the precise outline of the angulo-articular could not be seen in the conventional X-rays. Our analysis confirms the observations of Campos-da-Paz [28] and additionally demonstrates that *Tembeassu* has an angulo-articular with anterior limb longer than the posterior one (Figs 5 and 6). We, therefore, corroborate the hypothesis that *T*. *marauna* does not belong to the *Apteronotus s*.*s*. and, given the two possible scenarios of phylogenetic allocation of this genus (Fig 11), *Tembeassu* should be maintained as valid.

### The mandibular lobe in the Apteronotidae

**Fig 13:**
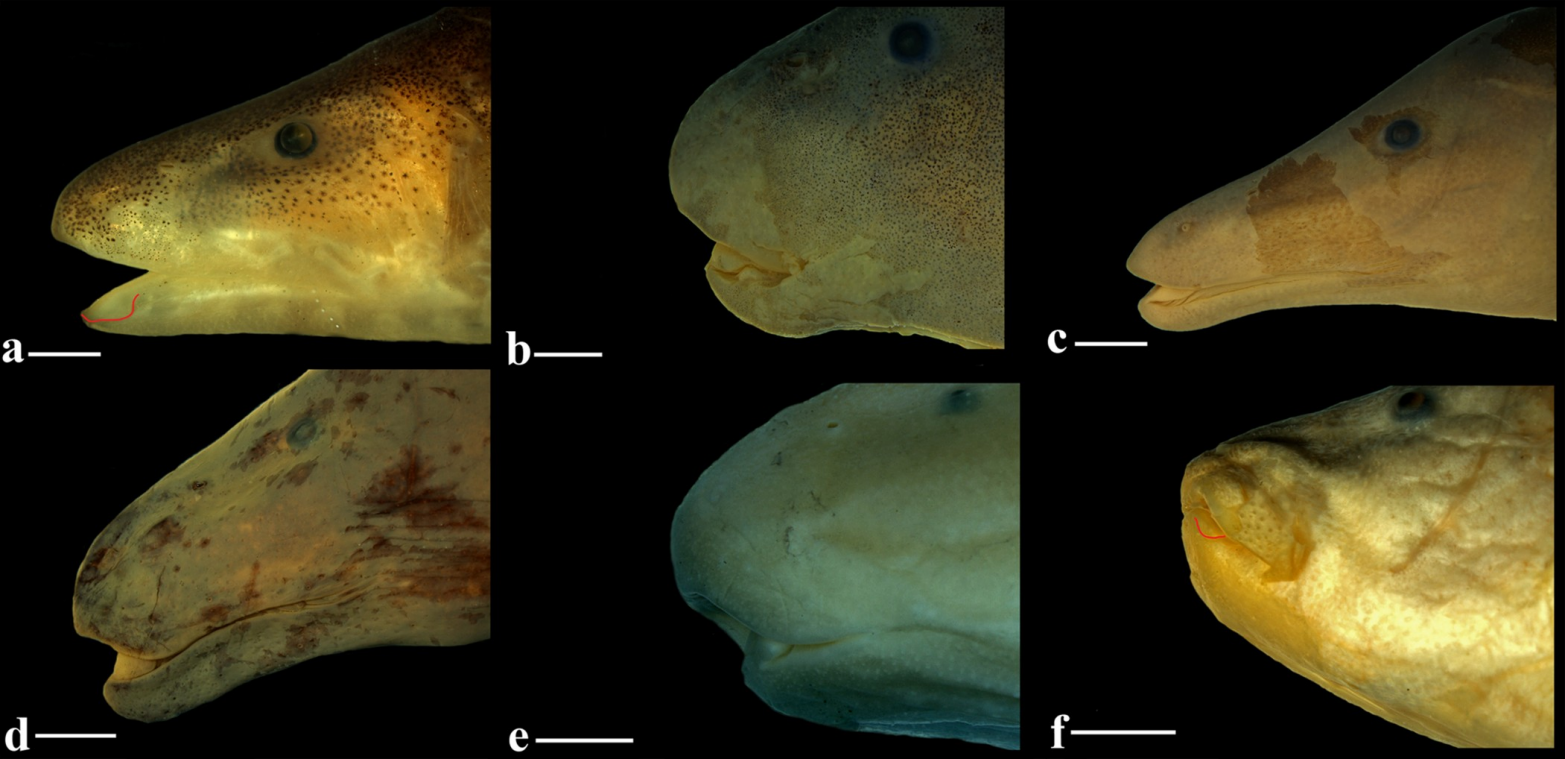
Close up of preorbital region of some apteronotids. (A) *Compsaraia compsa*, MZUSP 56543, 24.7 mm HL. (B) “*Sternarchella*” *curvioperculata*, MZUSP 23099, 26.6 mm HL. (C) *Apteronotus acidops*, MZUSP 22944, 33.1 mm HL. (D) *Apteronotus lindalvae*, MZUSP 120409, 34.3 mm HL. (E) *Pariosternarchus amazonenses*, MZUSP 48854, 32.9 mm HL. (F) *Sternarchella terminalis*, MPEG 3481, 28.5 mm HL. Skin flap or lateral chin lobe highlighted by a red line in A and F.

In the original description of *Tembeassu*, Triques [25] proposed the “enlarged fleshy lateral lobe on chin” as the unique diagnostic character for the genus. That structure is hypothesized to be derived from an expansion in the skin flap present at the anterior portion of the lower jaw of all apteronotids [25,28]. Our comparative sampling of gymnotiforms corroborates that hypothesis, but reveals the existence of a number of alternative morphologies concerning that mandibular skin flap, with some apteronotids exhibiting moderately developed fleshy lateral lobes on the chin (Fig 13). In *Orthosternarchus* Ellis, 1912 and *Sternarchorhamphus* Eigenmann, 1905, for instance, the skin flap is poorly differentiated from the surrounding epithelium and there is no lobe on its anterior portion. A similar condition, but with a somewhat more developed skin flap, is found in *Adontosternarchus*, *Sternarchorhynchus* and *Platyurosternarchus* Mago-Leccia, 1994. *Parapteronotus* Albert, 2001 have a thin skin flap with a U-shaped fold on its anteriormost portion. That morphology is basically the same for *Compsaraia*, although the skin fold is slightly trapezoidal in this genus (Fig 13A). In *Porotergus* Ellis, 1912 and “*Sternarchella*” *curvioperculata* Godoy (an apteronotid species currently with uncertain status; see Triques [20] and Ivanyisky & Albert [55]) the mandibular lobe is undifferentiated, but the skin flap is thicker at its anteriormost region (Fig 13B). A conspicuous presentation of the mandibular lobe occurs, besides *Tembeassu*, in *Apteronotus sensu lato* [4] (Fig 13C and 13D), *Megadontognathus*, *Melanosternarchus*, *Pariosternarchus* (Fig 13E), *Sternarchella* (Fig 13F) and *Sternarchogiton*. In these genera, the mandibular lobe varies in size and shape (from fusiform to teardrop-like). Nevertheless, in all conditions described above, the mandibular lobe is relatively small (see measurements in Diagnosis) visible only in lateral view, thus differing significantly from the hypertrophied lobe visible in ventral view in *Tembeassu*. Therefore, as interpreted herein, the hypertrophied condition (rather than the presence of the mandibular lobe) constitutes an autapomorphy for *T*. *marauna*. Given the highly conflicting hypotheses of relationships within Apteronotidae [4, 27, 31, 32, 38, 56], the evolution of the mandibular lobe in the family remains uncertain.

### Extra oral teeth in Gymnotiformes

**Fig 14:**
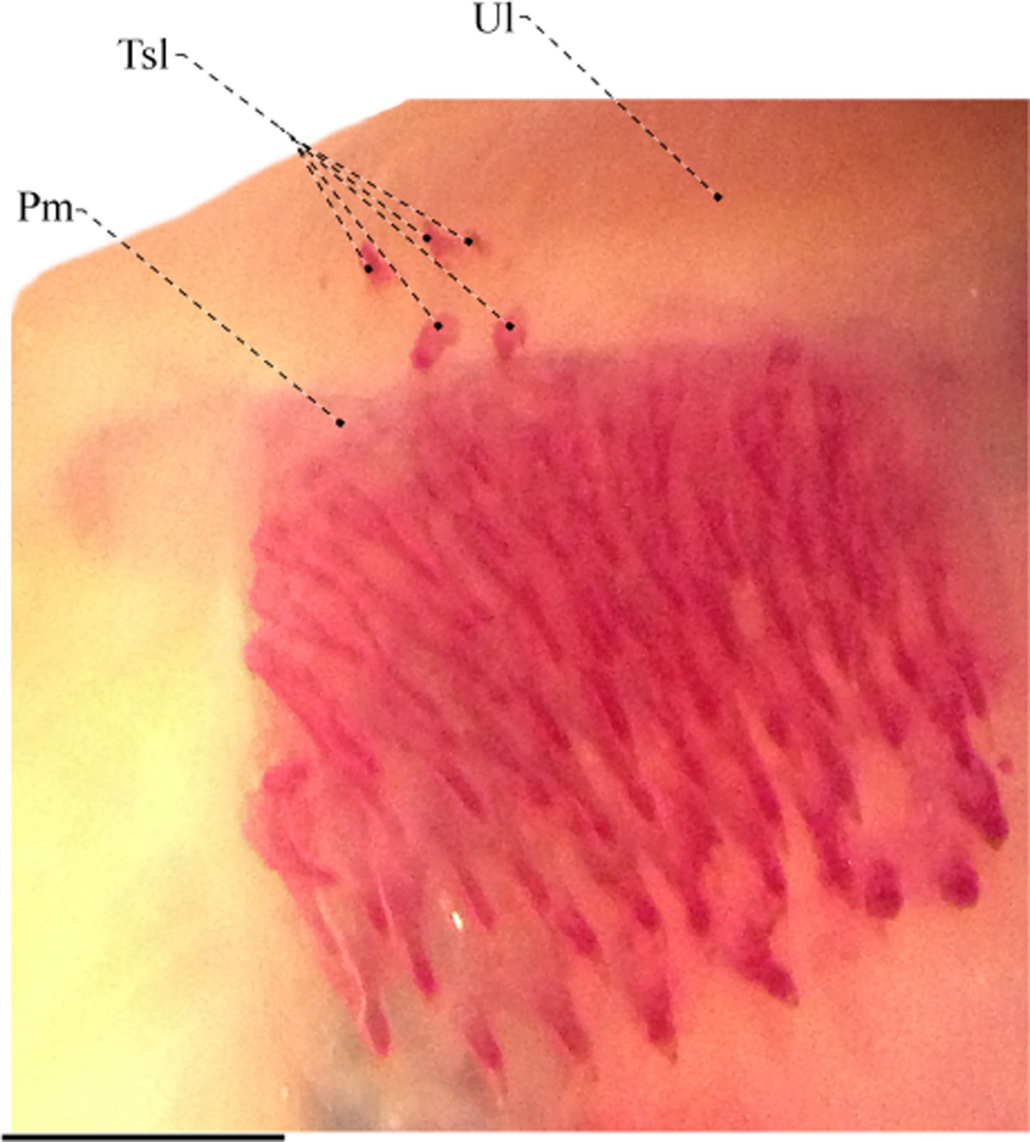
Ventral view of upper lip of *Distocyclus guchereauae*, MNHN 2003–0013, 222.0 mm *T*_L_, paratype.

The patch of extra teeth on the upper lip occurs in all known specimens of *Tembeassu*, two males and one female, apparently not related to sexual dimorphism, and its occurrence could be related to feeding habits [28]. Among gymnotiforms, only the sternopygids *Japigny kirschbaumi* Meunier, Jégu & Keith and *Distocyclus guchereauae* Meunier, Jégu & Keith also possess extra teeth on the upper jaw (Fig 14). However, the extra teeth observed in these genera differ in position, morphology, and form of attachment compared to *Tembeassu*.

In *Tembeassu*, there are 15–22 closely set rostral teeth directly implanted into the soft tissue of the upper lip anterior to the premaxilla, and all teeth are conical, slender, with posteriorly curved tip (Figs 5 and 6). In *Japigny*, the extra teeth occur in the upper oral valve, lying posteroventral to the premaxilla (*pers*. *obs*.; [57]), and with a tooth morphology similar to the villiform teeth in the premaxilla and dentary. In *D*. *guchereauae*, five villiform and well-separated extra teeth are arranged in two rows located just anterior to the premaxilla (Fig 14). We were unable to infer if the extra teeth in these sternopygid genera are sexually dimorphic or related to feeding habits due the reduced number of specimens available for dissections. *Japigny* and *Distocyclus* appear far removed from each other in the sternopygid tree [31, 57], whereas *Tembeassu* is well nested within Apteronotidae. Therefore, the possession of extra teeth in the upper jaw is most parsimoniously optimized as having homoplastically evolved in each genus.

## Supporting information

S1 File Data matrix employed in the present analysis in TNT format. (TNT)

S2 File Strict consensus of 99,999 most parsimonious trees (586 steps) obtained in the maximum parsimony analysis. (EMF)

S3 File Majority-rule consensus (B) of 99,999 most parsimonious trees (586 steps) obtained in the maximum parsimony analysis. (EMF)

S4 File Strict consensus of 42,566 MPT’s (586 steps) resolving *Tembeassu marauna* as the sister group to all remaining apteronotids. (EMF)

S5 File Strict consensus of 57,433 MPT’s (586 steps) resolving *Tembeassu marauna* as the sister group to the clade *Megadontognathus* + *Apteronotus s*. *s*. (EMF)
